# Development of a CRISPR/Cas9-mediated gene-editing method to isolate a mutant of the unicellular green alga *Parachlorella kessleri* strain NIES-2152 with improved lipid productivity

**DOI:** 10.1186/s13068-024-02484-7

**Published:** 2024-03-05

**Authors:** Yuki Kasai, Satsuki Takagi, Shuhei Ota, Kotaro Ishii, Tsuyoshi Takeshita, Shigeyuki Kawano, Shigeaki Harayama

**Affiliations:** 1 Research and Development Initiative, Chuo University, Bunkyo-Ku, Tokyo, 112-8551 Japan; 2 Department of Integrated Biosciences, Graduate School of Frontier Sciences, University of Tokyo, 5-1-5 Kashiwanoha, Kashiwa, Chiba 277-8562 Japan; 3 Department of Radiation Measurement and Dose Assessment, National Institutes for Quantum Science and Technology, 4-9-1 Anagawa, Inage-Ku,, Chiba-Shi, 263-8555 Japan; 4 Present Address: Department of Biological Science, Chuo University, Kasuga 1-13-27, Bunkyo-Ku, Tokyo, 112-8551 Japan; 5 Biodiversity Division, National Institute for Environmental Studies, Tsukuba, Japan

**Keywords:** *Parachlorella kessleri*, Genetic transformation, Electroporation, Genome editing, CRISPR/Cas9

## Abstract

**Background:**

Previously, we isolated a mutant of *Parachlorella kessleri* named strain PK4 that accumulated higher concentrations of lipids than the wild-type strain. Resequencing of the PK4 genome identified mutations in three genes which may be associated with the high-lipid phenotype. The first gene, named *CDMT1*, encodes a protein with a calcium-dependent membrane association domain; the second gene, named *DMAN1*, encodes endo-1,4-β-mannanase, while the third gene, named *AATPL1*, encodes a plastidic ATP/ADP antiporter-like protein.

**Results:**

To determine which of these mutant genes are directly responsible for the phenotype of strain PK4, we delivered Cas9-gRNA ribonucleoproteins targeting each of the three genes into the wild-type cells by electroporation and successfully disrupted these three genes separately. The lipid productivity in the disruptants of *CDMT1* and *DMAN1* was similar to and lower than that in the wild-type strain, while the disruptants of *AATPL1* exhibited > 30% higher lipid productivity than the wild-type strain under diurnal conditions.

**Conclusions:**

We succeeded in improving the lipid productivity of *P. kessleri* by CRISPR/Cas9-mediated gene disruption of *AATPL1*. The effective gene-editing method established in this study will be useful to improve *Parachlorella* strains for industrial applications.

**Supplementary Information:**

The online version contains supplementary material available at 10.1186/s13068-024-02484-7.

## Background

*Parachlorella kessleri* is a unicellular alga belonging to the family Chlorellaceae in the class Trebouxiophyceae, with a haploid life cycle characterized by autospore formation [1]. This species exhibits a relatively rapid photosynthetic growth rate and accumulates a high level of lipids under stress conditions [2, 3], making it a top contender for biodiesel production. Furthermore, *P. kessleri* serves as excellent feedstock for high-value products, including carotenoids exhibiting antioxidant activities [4] and extracellular polysaccharides (EPS) exhibiting antiproliferative and immune-modulatory activities as well as heavy metal sorption ability [5–7]. Moreover, this organism demonstrates efficient nitrogen and phosphorous removal from wastewater [8, 9]. Due to these features, *P. kessleri* has garnered considerable interest across various industrial sectors. Formerly known as *Chlorella kessleri* [1, 10, 11], *P. kessleri* has been utilized as a food supplement for many years, gaining recognition as a safe organism [12, 13]. Consequently, this organism is considered suitable for outdoor mass cultivation for the production of biofuel, food, feed, other high-value products, and wastewater treatment.

Despite the immense potential of *P. kessleri* for industrial applications, the production costs associated with raw materials from this alga are still too high to be competitive with existing materials. Accordingly, significant cost reduction in manufacturing biomass and value-added products is needed. One method to realize this is strain improvement, achieved through molecular breeding. A draft genome sequence of *P. kessleri* strain NIES-2152, crucial information for molecular breeding, has been determined, and major metabolic pathways have been annotated [14, 15]; GenBank assembly accession number: GCA_001598975. Furthermore, interesting mutants have been isolated through mutagenesis by heavy-ion-beam irradiation [16, 17]. One such mutant named strain PK4 exhibited higher levels of lipid accumulation, and whole-genome resequencing of this strain identified mutations in three genes, 9934_t (GenBank accession number LC424333), 8741_t (LC42335), and 9067_t (LC424334) [14, 17]. The 9934_t gene was deduced to code for a protein containing the C2 domain (Additional file 1: Figure S1a). This domain is known to be involved in calcium-dependent membrane targeting [18]. Thus, this gene is named *CDMT1*. The 8741_t gene, which consists of internally duplicated coding sequences of endo-1,4-β-mannanase, carries two conserved cellulase domains at its C-terminal half (Additional file 1: Figure S1b). We named this gene *DMAN1* (duplicated mannanases 1). The product of the 9067_t gene exhibited similarities to plastidic ATP/ADP translocases (AATPs) from *Arabidopsis thaliana*. Therefore, the gene was designated as *AATPL1* (*ATTP-like 1*). AATP is also called plastidic ATP/ADP antiporter or plastidic nucleotide transporter (NNT). The AATP family proteins are found in many plants and algae, localized to the inner membrane of the plastid envelope, and catalyze the import of ATP in the plastid coupled with the export of ADP from the plastid. AATPs provide the plastid stroma with ATP, required for many anabolic processes [19]. The AATP family generally comprises 12 transmembrane helices [20–22] (Additional file 1: Figure S1c). In the protein sequence of AATPL1, 12 transmembrane helices were predicted using the Deep TMHMM program [23], with 6 helices located in the N-terminal half and another 6 helices located in the C-terminal half. However, from the gene annotations, we were unable to identify which of these mutations was responsible for the high-lipid phenotype.

Then, we became interested in developing CRISPR-based gene-editing tools because editing the three genes, *DMAN1*, *CDM1*, and *AATPL1*, individually would help identify the causative mutation responsible for the high lipid accumulation in strain PK4. One modern CRISPR tool involves the direct delivery of a ribonucleoprotein (RNP) complex consisting of Cas9 protein and guide RNA (gRNA). However, the application of this new technology requires effective delivery methods. Thus far, two methods for delivering DNA (genetic transformation) into *P. kessleri* cells have been reported. In one study, the spectinomycin-resistant gene was introduced using biolistic bombardment, and the integration of the gene into the chloroplast genome via homologous recombination was achieved at a frequency of 1–2 × 10^−6^ per input cell [24]. In another study, the hygromycin-resistant gene was integrated into the nuclear genome via *Agrobacterium*-mediated transformation at a frequency of 2.5 × 10^−5^ per input cell [25].

Recently, electroporation has become a preferred method for introducing biomolecules such as polynucleotides and proteins into algal cells [26–30] due to its easier operation compared to other methods. In the present study, we developed electroporation-based genetic transformation and gene-editing methods applicable to *P. kessleri*, and these methods were used to knockout *DMAN1*, *CDM1,* and *AATPL1*. From the phenotype analyses of the knockout mutants, it was concluded that the inactivation of *AATPL1* provoked high lipid accumulation.

## Results and discussion

### Synchronization of cultures of strain NIES-2152

**Fig. 1:**
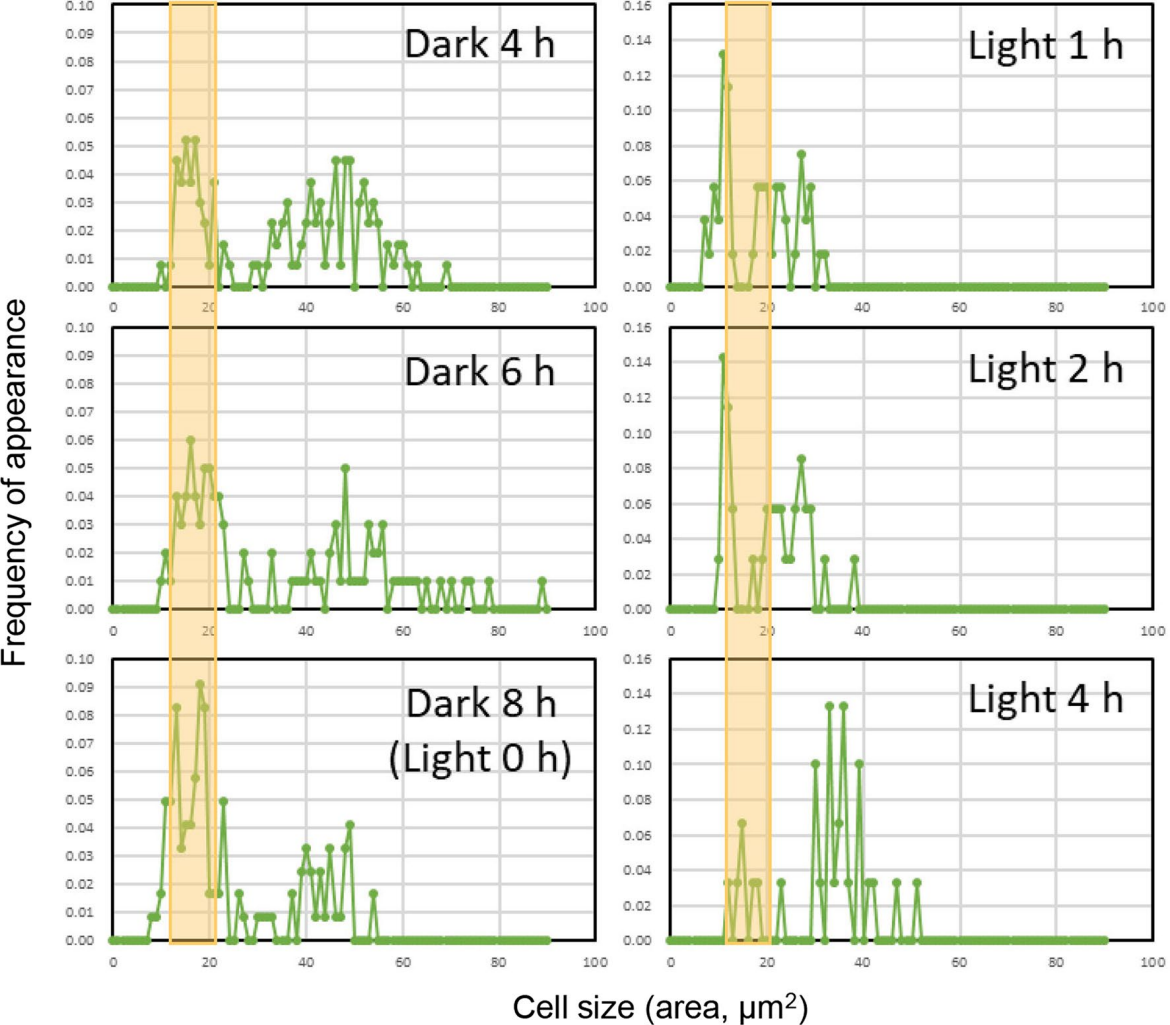
Changes in cell size of strain NIES-2152 under diurnal rhythms. Cells of strain NIES-2152 were cultured in BG-11 medium under the L/D 16:8 cycle. Samples were taken at different time points during the 3rd dark period and 4th light period. The horizontal axis represents the cell area (μm^2^) determined using the ImageJ software, while the vertical axis represents the frequency of appearance

In our previous study on the development of a genetic transformation system applicable to the autospore-forming green alga *Coccomyxa*, we discovered that young daughter cells, released from cleaved mother cell walls, exhibit high competence to receive transforming DNA [30]. *P. kessleri* strain NIES-2152 (hereinafter referred to as "strain NIES-2152" or "the wild-type strain") is also an autospore-forming unicellular green alga, and it has been reported that the cell wall thickness is thinnest in daughter cells immediately after their release from their mother cell walls [1]. To enrich such daughter cells, cells of strain NIES-2152 were grown under a (16-h light)/(8-h dark) cycle (L/D 16:8 cycle) for 3–6 days to synchronize nuclear and cellular division. Light-microscopic observation of the cells revealed that the cell population with smaller cell sizes increased with an increased dark period (Additional file 2: Figure S2). The percentage of cells with a cross-sectional area lower than 20 μm^2^ reached its maximum during the cycle between 8-h dark (= 0-h light) and 1-h light (Fig. 1). Subsequently, cell sizes increased with an increased light period. This observation indicated that the cleavage of the mother-cell wall, followed by the release of young daughter cells, terminated around the end of the dark period.

### Construction of plasmids for genetic transformation

To express two drug-resistant genes, the bleomycin/phleomycin/zeomycin (Zeocin^®^) resistant gene (*ble*) and the neomycin/G418 resistant gene (*neo*), in strain NIES-2152 as selection markers, effective promoters and terminators are required. For this purpose, highly expressed genes in strain NIES-2152 were selected from our preliminary transcriptome data, and their 5ʹ-untranslated region (UTR) and the 3ʹ-UTR were defined as putative promoter and terminator regions. Consequently, the heat shock protein 90-family gene (*HSP90*) and the plastidic ATP/ADP translocase 1 gene (*AATP1*) were chosen as they possess the promoter and terminator regions that could be confined within 1-kb regions. Additionally, the promoter/terminator regions from the ribulose-1,5-bisphosphate carboxylase/oxygenase small subunit gene 4 (*RBCS4*) were used because the promoters and terminators of *RBCS* from various green algae including *Chlamydomonas reinhardtii* [31–36], *Coccomyxa* sp. [37], and *Dunaliella salina* [38], were successfully used for expressing transgenes. The promoter and terminator regions of these three genes were PCR-amplified and fused to the coding sequences of the *ble*, *neo*, or codon-optimized *neo* genes.

### Genetic transformation of strain NIES-2152 by electroporation

In antibiotic susceptibility tests, no spontaneous Zeocin®-resistant (Zeo^r^) mutants of strain NIES-2152 were observed when 10^7^ cells were spotted on agar plates containing 35 μg ml^−1^ Zeocin^®^ (Additional file 3: Figure S3a). Zeo^r^ transformants were then screened on agar plates containing 35 μg ml^−1^ Zeocin® after the introduction of bleHH, an expression construct of *ble* with the promoter and terminator sequences of *HSP90* (Additional file 4: Figure S4), using an ELEPO21 electroporator, which delivers two types of electric pulses called the poring pulse (Pp) and the transfer pulse (Tp) (https://www.nepagene.jp/e_products_nepagene_0029.html). To optimize electroporation conditions, the electric-field strength and pulse width of Pp were changed between 1500 and 2500 V cm^−1^, and between 2.5 and 15 ms, respectively. For Tp, the electric-field strength was changed between 100 and 500 V cm^−1^, while the pulse width and number of pulses were fixed at 50 ms and 5 pulses, respectively. The cells of strain NIES-2152 were cultivated in BG-11 medium under an L/D 16:8 cycle and harvested 2 h after the beginning of the light period when OD_750_ ranged between 0.2 and 2. A total of 114 electroporation runs were conducted using eight independent cultures under various combinations of electric-field strengths and pulse widths, and electroporated cells were subsequently selected for growth on agar plates containing Zeocin^®^ to obtain Zeo^r^ colonies as described in ``Methods’’ section. The presence of the *ble* coding sequence in the genomes of Zeo^r^ colonies (Additional file 3: Figure S3b) isolated from 13 independent electroporation samples was examined by PCR. All 116 Zeo^r^ transformants examined were positive in the *ble* PCR (Additional file 5: Figure S5). Accordingly, genetic transformation frequencies were calculated by dividing the number of Zeo^r^ colonies on the selection plates by the number of input cells.

**Fig. 2:**
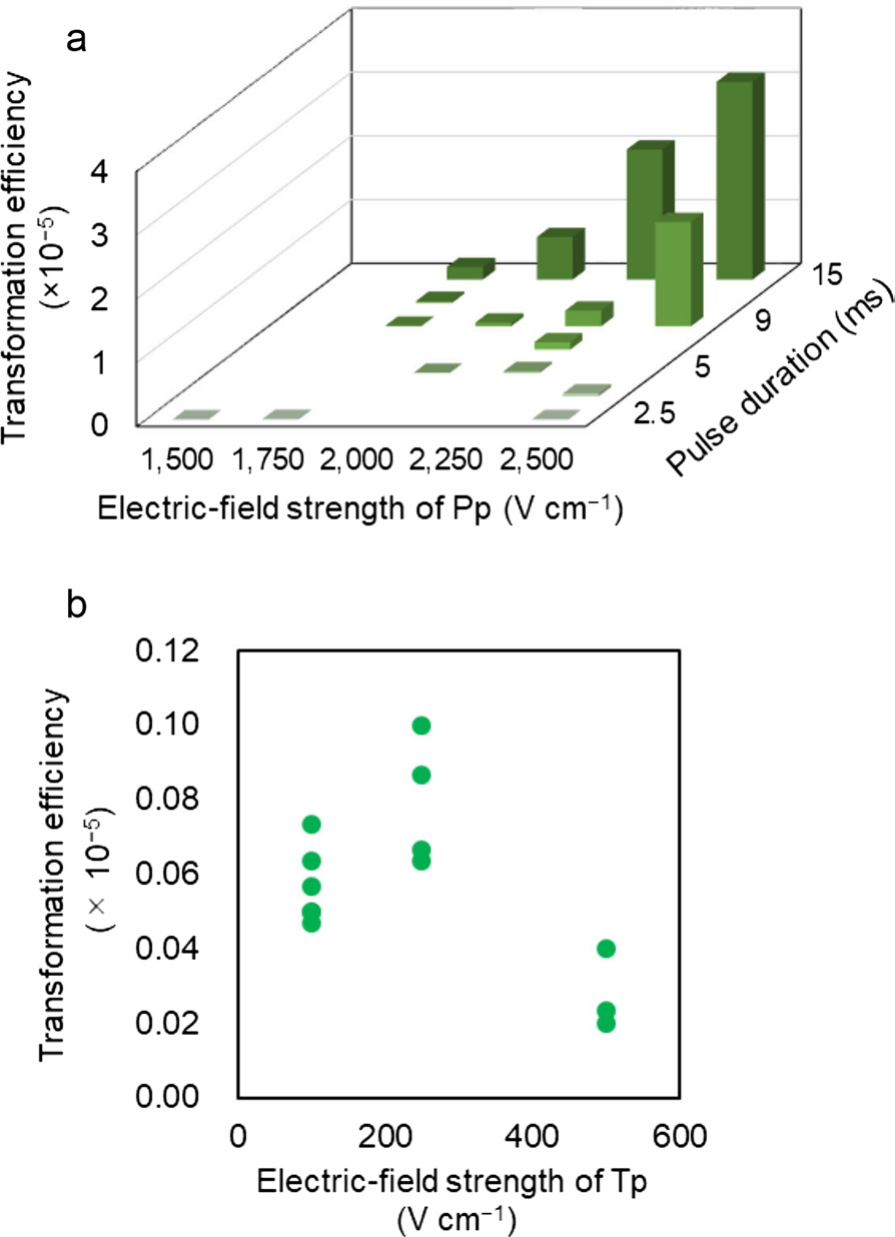
Transformation efficiency of strain NIES-2152. **a** Transformation efficiencies at different electric-field strengths with various pulse durations of Pp. The electric-field strength, number of pulses, and pulse duration of Tp were fixed at 100 V cm^−1^, 5 pulses, and 50 ms, respectively. **b** Effect of electric-field strength of Tp on transformation efficiency. The electric-field strength and pulse duration of Pp were fixed at 2000 V cm^−1^ and 9 ms, respectively. The transformation efficiency was calculated by dividing the number of Zeocin^Ⓡ^-resistant colonies by the number of input cells (3.0 × 10^7^)

The transformation frequencies at different electric-field strengths of Pp were shown in Fig. 2a. The results demonstrated that the highest transformation efficiency was achieved when the electric-field strength and pulse width of Pp were set at 2,500 V cm^−1^ and 15 ms, respectively. To determine the optimum Tp conditions, the electric-field strength and pulse width of Pp were fixed at 2000 V cm^−1^ and 9 ms, respectively, while three electric-field strengths of Tp at 100, 250, or 500 V cm^−1^ were tested (Fig. 2b). The results demonstrated that an electric-field strength of Tp at 250 V cm^−1^ achieved the highest transformation efficiency.

The effect of growth stage on transformation efficiency was also examined as follows. Cells were harvested at OD_750_ of 0.5 or 1.2, and electroporation was conducted with Pp electric-field strength of 2500 V cm^−1^, Pp pulse width of 9 ms, and Tp electric-field strength of 100 V cm^−1^. The transformation efficiency of cells harvested at OD_750_ of 0.5 was 1.4 ± 0.6 × 10^−5^ transformants per input cell, while that harvested at OD_750_ of 1.2 was 5.2 ± 0.4 × 10^−6^ transformants per input cell. Under other electroporation conditions as well, cells harvested at OD_750_ below 1 showed higher transformation efficiency than those harvested at OD_750_ above 1. This led us to conclude that the transformation efficiency is higher in cells harvested at OD_750_ less than 1. The highest genetic transformation efficiency, ranging from 3.2 to 4.6 × 10^−5^ transformants per input cell, was obtained under the following conditions: Pp electric-field strength of 2500 V cm^−1^, Pp pulse width of 15 ms, Tp electric-field strength of 250 V cm^−1^, and harvesting cell density less than OD_750_ of 1.

When cells grown to OD_750_ between 0.2 and 0.5 were harvested at 0, 1, and 2 h after the beginning of the light period, and electroporated with Pp at 2500 V cm^−1^ for 15 ms, and Tp at 250 V cm^−1^, the transformation efficiencies were 3.8 ± 0.1 × 10^−5^, 2.3 ± 0.8 × 10^−5^, and 4.0 ± 0.6 × 10^−5^ transformants per input cell, respectively. Thus, no statistical difference was observed between the transformation efficiencies of cells harvested at different time points between 0 and 2 h after the onset of the light period. Similar results were obtained under other electroporation conditions.

We also conducted 30 independent electroporations using cells grown under continuous light, i.e., without synchronization. The average transformation efficiency was only 3.5 ± 3.8 × 10^−7^ transformants per input cell, which was two orders of magnitude smaller than that obtained with synchronized cells. Therefore, synchronization was found to be crucial for efficient genetic transformation in strain NIES-2152.

Finally, we compared the transformation efficiencies of bleHH with those of other two *ble* expression constructs, bleAA and bleRR, consisting of the promoter and terminator sequences of *AATP1* and *RBCS4*, respectively, flanking the *ble* coding sequence, using the optimum electroporation conditions described above. With each of these constructs, 10 electroporation experiments were carried out. The transformation efficiencies with bleHH, bleRR, and bleAA were 2.5 ± 1.4 × 10^−5^, 2.8 ± 0.6 × 10^−5^, and 2.0 ± 1.0 × 10^−6^ transformants per input cell, respectively. The size of bleAA is almost the same as that of bleHH, and smaller than that of bleRR; therefore, the low transformation efficiency of bleAA should not be due to its size.

The *neo* gene has been commonly used as a selectable marker for the introduction of genes into plant cells. Therefore, we next analyzed the utility of the *neo* gene as a selectable marker in *P. kessleri*. No spontaneous G418-resistant (G418^r^) mutant were obtained when 10^7^ cells of strain NIES-2152 were spotted on agar plates containing 25 μg ml^−1^ G418 (Additional file 3: Figure S3a). Therefore, transformants were screened on agar plates containing 25 μg ml^−1^ G418 after the introduction of the *neo* expression construct, neoHH, using the optimized electroporation conditions described above. However, no G418^r^ transformant was obtained with occasional spontaneous G418^r^ colonies in 7 independent electroporation experiments. Then, a codon-optimized *neo*-expression construct, PkneoHH, was constructed as described in “Methods” section and introduced into cells. In 8 independent experiments, a total of 48 G418^r^ colonies were isolated, among which only four G418^r^ colonies were positive in the *neo* PCR. When G418 concentration was raised to 30 μg ml^−1^, no colonies were formed on the plates. From these results, we concluded that the *neo* gene is practically not useful as a selection marker for this strain.

### Gene knockout by CRISPR/Cas9

Previously, we successfully delivered Cas9–gRNA complex into *Coccomyxa* cells using conditions optimal for genetic transformation of this organism [27]. Based on this previous result, we delivered Cas9-RNP into cells of strain NIES-2152 under conditions optimal for genetic transformation of this strain to disrupt three genes, *CDMT1*, *DMAN1*, and *AATPL1*.

**Fig. 3:**
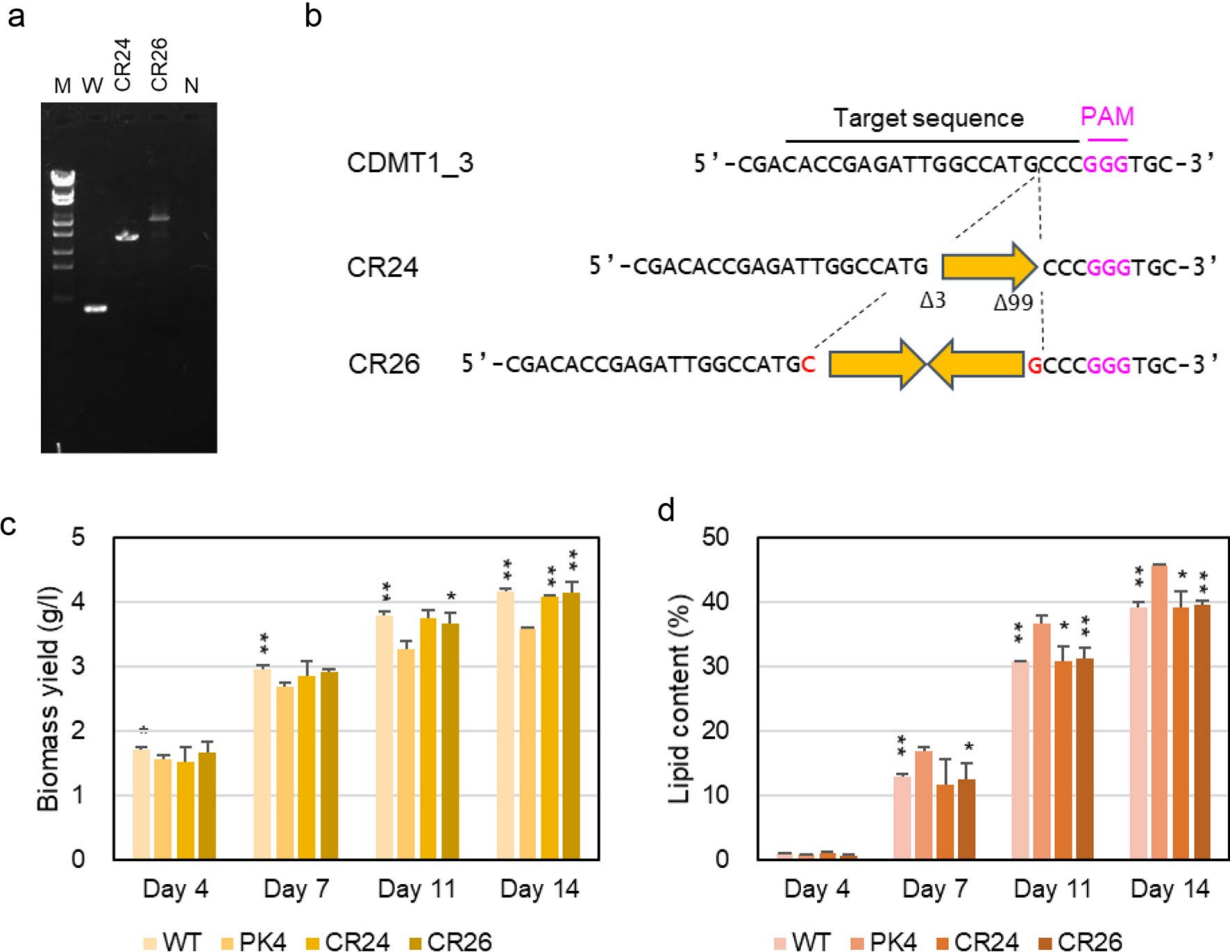
Construction and characterization of *CDMT1*-knockout strains. **a** PCR detection of a bleHH insertion into a crRNA target site. M: molecular size markers (λ-EcoT14 I digest), PCR product amplified with DNA from W: strain NIES-2152, CR24: strain CR24, CR26: strain CR26, and N: no DNA. **b** The target sequence of CDMT1_3 crRNA and the corresponding sequences in the knockout strains, CR24 and CR26. Each broad orange arrow represents one unit length of bleHH. A Δ symbol followed by a number indicates a deletion of bases with this number at one end of bleHH. Inserted nucleotide(s) are shown in red. **c**, **d** Cells of the wild-type strain, strain PK4, and *CDMT1*-knockout strains were grown in 1/5 UP medium under continuous light for 4 to 14 days. **c** Volumetric biomass yield [cell mass dry weight per liter of culture (g l^−1^)]. **d** Lipid content in percentage of dry weight biomass (w/w). Bars represent standard deviations of data from three independent samples. Statistical significance of differences between the values of strain PK4 and those of other strains were tested by Student’s t test (two-tailed), and the results are shown as asterisks. Single asterisks indicate P values between 0.01 and 0.05, and double asterisks indicate *P* < 0.01

To disrupt *CDMT1*, three CRISPR RNAs (crRNAs) targeting the C2 domain, named CDMT1_1, CDMT1_2, and CDMT1_3 (Additional file 7: Table S2, Additional file 1: Figure S1a), were designed. Each gRNA containing the crRNAs was conjugated with Cas9 protein, and the RNP complex and bleHH were delivered into cells of strain NIES-2152. DNA was isolated from 7, 13, and 48 Zeo^r^ colonies isolated after introducing the CDMT1_1, CDMT1_2, and CDMT1_3 gRNAs, respectively. Since the detection of base-substitution and small indel mutations is cumbersome and requires time-consuming analysis, we only surveyed Zeo^r^ transformants carrying a knock-in of the *ble* gene at targeted sequences. Clones containing a bleHH insertion in the crRNA recognition sequence were screened by PCR using gene-specific primer sets, CDMT1_F and CDMT1_R (Additional file 7: Table S1), and bleHH insertions were detected only in the CDMT1_3 target (target 3) site. The gene knock-in frequency was 7.2 × 10^−7^ per input cell. Two independently isolated clones, CR24 and CR26, each carrying a bleHH insertion at the target 3 site, were selected for further analyses. DNA sequencing of the target 3 region of strain CR24 revealed a truncated bleHH fragment with a 3-bp deletion (Δ3) and a 99-bp deletion (Δ99) at the 5ʹ- and 3ʹ-sides of bleHH, respectively, which was inserted at the position 3-bp upstream of the protospacer adjacent motif (PAM) site where Cas9 recognizes and cleaves DNA (Fig. 3b). On the other hand, the target site of strain CR26 cleaved 3-bp upstream of the PAM site acquired 1-bp insertions at both ends of the cleavage site, between which two copies of bleHH in an inverted orientation were integrated (Fig. 3b).

**Fig. 4:**
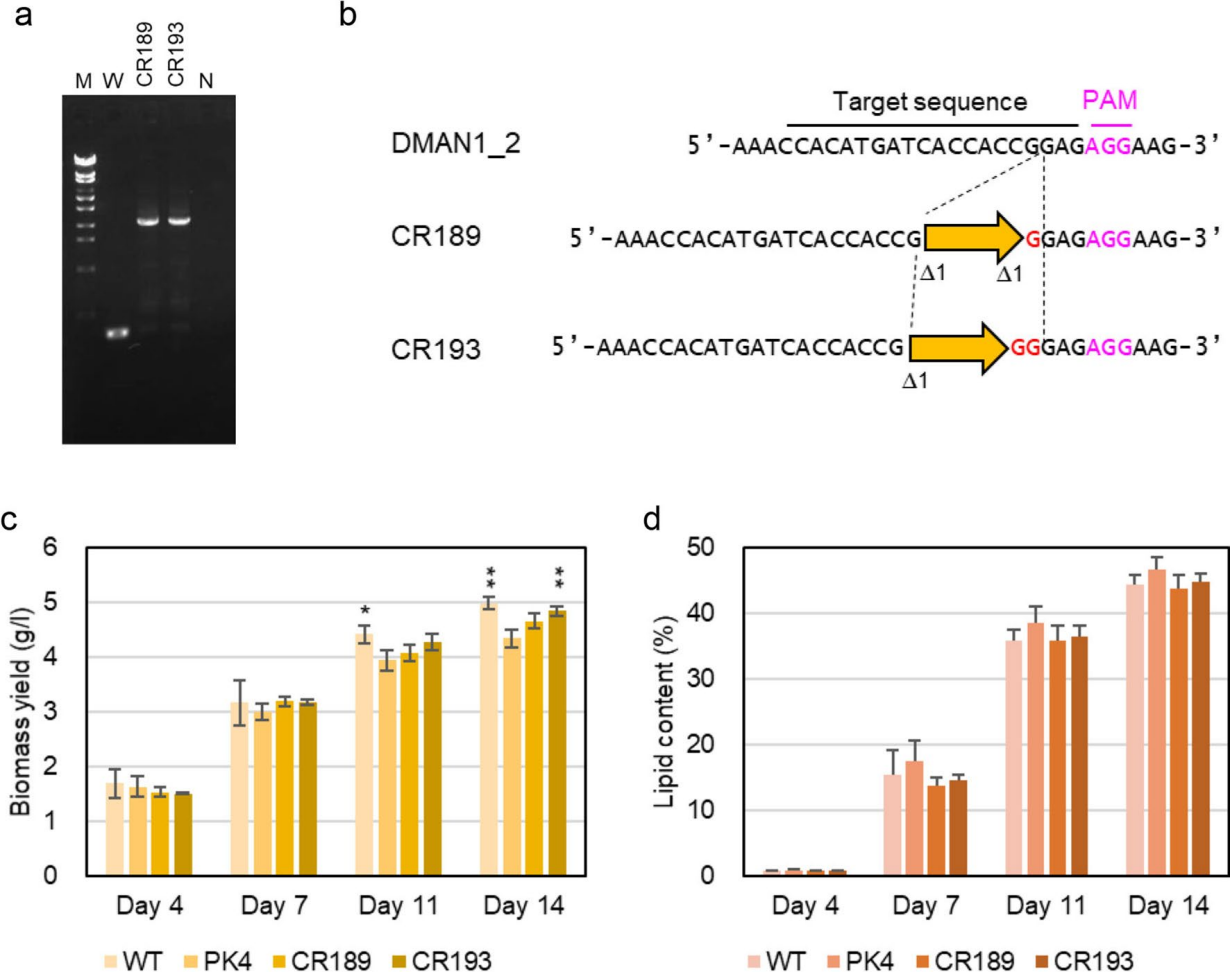
Construction and characterization of *DMAN1*-knockout strains. **a** PCR detection of a bleHH insertion. The primer set DMAN1_F and DMAN1_R2 (Additional file 7: Table S1) was used to detect bleHH insertion into a crRNA target site. M: molecular size markers (λ-EcoT14 I digest), PCR product amplified with DNA from W: strain NIES-2152, CR189: strain CR189, CR193: strain CR193, and N: no DNA. **b** The target sequence of DMAN1_2 crRNA and the corresponding sequences in the knockout strains, CR189 and CR193. Each of broad orange arrow represents one unit length of bleHH. A Δ symbol followed by a number indicates a deletion of base(s) with this number at one end of bleHH. Inserted nucleotide(s) are shown in red. **c**, **d** Cells of the wild-type strain, strain PK4, and *CDMT1*-knockout strains were grown in 1/5 UP medium under continuous light for 4–14 days. **c** Volumetric biomass yield [cell mass dry weight per liter of culture (g l^−1^)]. **d** Lipid content in percentage of dry weight biomass (w/w). Bars represent standard deviations of data from three independent samples. Statistical significance of differences between the values of strain PK4 and those of other strains were tested by Student’s t test (two-tailed), and the results are shown as asterisks. Single asterisks indicate P values between 0.01 and 0.05, and double asterisks indicate *P* < 0.01

Next, we disrupted *DMAN1*. Three different sequences within the cellulase domain were targeted using designed crRNAs named DMAN1_1, DMAN1_2, and DMAN1_3 (Additional file 7: Table S2, Additional file 1: Figure S1b). Each gRNA containing the crRNAs was conjugated with Cas9 protein, and the RNP complex and bleHH were delivered into cells of strain NIES-2152. DNA was isolated from 40, 40, and 37 Zeo^r^ colonies obtained after introducing the DMAN1_1, DMAN1_2, and DMAN1_3 gRNAs, respectively. Clones containing a bleHH insertion in the crRNA recognition sequence were screened by PCR using the primer set, DMAN1_F and DMAN1_R (Additional file 7: Table S1). From most of the clones, the 1492-bp-long DNA corresponding to the PCR product from the wild-type sequence was amplified, except for three DMAN1_2 targeted clones from which longer DNA fragments of about 3 kb were amplified in addition to the 1492-bp-long DNA fragment (Additional file 6: Figure S6). The gene knock-in frequency was 2.3 × 10^−7^ per input cell. This result suggested that these three clones were not pure but consisted of two types of populations, one carrying a bleHH insertion in the target site, and the other not carrying bleHH insertion in the target site. Therefore, these clones were further purified through several rounds of single colony isolation, and pure clones carrying a bleHH insertion in the target site were recovered from two of the three clones. The two clones were named strains CR189 and CR193, and their genomic DNA around the crRNA recognition sequence was sequenced. The DNA sequence in strains CR189 indicated that the target sequence had been cleaved 3-bp upstream of the PAM sequence into which bleHH with a 1-bp deletion (Δ1) at both sides and a 1-bp (G) insertion at one side was integrated (Fig. 4b). On the other hand, in the genome of strain CR193, one copy of bleHH with a 1-bp deletion (Δ1) at one side and a 2-bp (GG) insertion at the other side was integrated at the cleavage site (Fig. 4b).

**Fig. 5:**
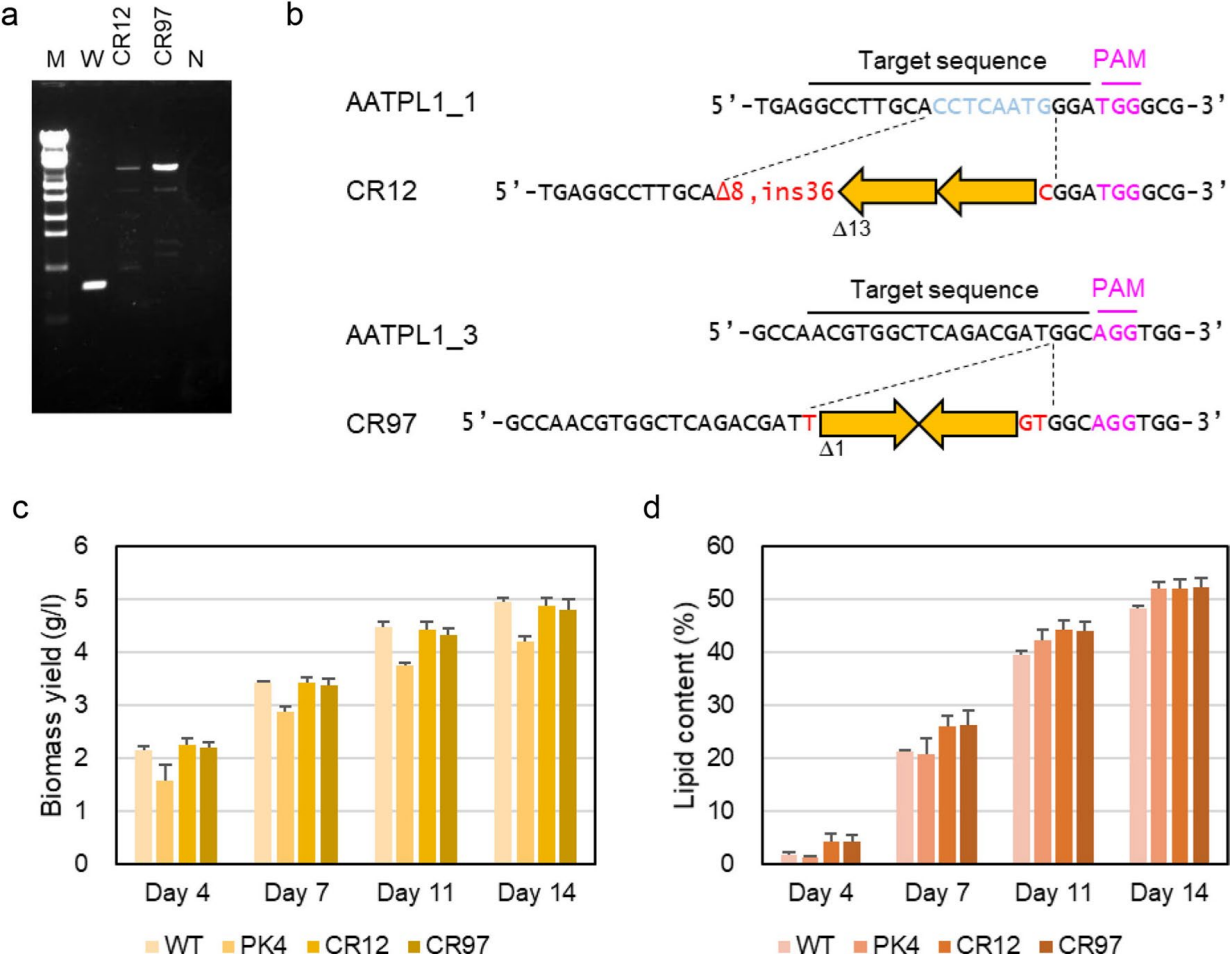
Construction and characterization of *AATPL1*-knockout strains. **a** PCR detection of a bleHH insertion into crRNA target sites. M: molecular size markers (λ-EcoT14 I digest), PCR product amplified with DNA from W: strain NIES-2152, CR12: strain CR12, CR97: strain CR97, and N: no DNA. **b** The target sequences of AATPL1_1- and AATPL1_3 crRNAs and the corresponding sequences in the knockout strains, CR12 and CR7. Each broad orange arrow represents one unit length of bleHH. A Δ symbol followed by a number indicates a deletion of base(s) with this number at one end of bleHH. Inserted nucleotide(s) are shown in red. At one side of the cleavage site generated by AATPL1_1 crRNA, an 8-bp deletion (Δ8) plus a 36-bp insertion (ins36) occurred. **c**, **d** Cells of the wild-type strain, strain PK4, and *AATPL1*-knockout strains were grown in 1/5 UP medium under continuous light for 4–14 days. **c** Volumetric biomass yield [cell mass dry weight per liter of culture (g l^−1^)]. **d** Lipid content in percentage of dry weight biomass (w/w). Statistical significance of differences between the values of strain PK4 and those of other strains were tested by Student’s t test (two-tailed), and the results are shown as asterisks. Single asterisks indicate *P* values between 0.01 and 0.05, and double asterisks indicate *P* < 0.01

Two conserved domains were predicted in the gene product of *AATPL1* by running InterProScan (IPR004667). We designed three crRNAs, namely AATPL1_1, AATPL1_2, and AATPL1_3, each targeting different sequences in the N-terminal IPR004667 domain (Additional file 7: Table S2, Additional file 1: Figure S1c). Each gRNA containing the crRNAs was conjugated with Cas9 protein, and the RNP complex and bleHH were delivered into cells of strain NIES-2152. Forty-five clones were randomly selected from Zeo^r^ transformants obtained with each of the three crRNAs for genomic DNA isolation, and those carrying a bleHH insertion in the crRNA recognition sequence were screened by PCR using the primer set, AATPL1_F and AATPL1_R (Additional file 7: Table S1). Knock-in clones were successfully obtained with all gRNAs. The gene knock-in frequency in the AATPL_1, AATPL_2, and AATPL3 target sites were 2.3, 1.5, and 6.9 × 10^−7^ per input cell, respectively. Two independent clones, CR12 and CR97, were then selected for further analyses. The DNA sequencing of the regions around the AATPL1_1 and AATPL1_3 target sites in the genomes of strains CR12 and CR97, respectively, revealed that their genomes have been cleaved 3-bp upstream of the PAM sequence followed by the insertion of bleHH. In strain CR12, two copies of bleHH were inserted in tandem, with a 13-bp deletion (Δ13) at one end. The target site was also rearranged after Cas9 cleavage: at one end, an 8-bp deletion (Δ8) plus a 36-bp insertion (ins36) were observed, while at the opposite end, a 1-bp insertion (C) occurred (Fig. 5b). In strain CR97, two copies of bleHH were inserted in inverted repeats with a 1-bp deletion (Δ1) at one end. The target site was also rearranged after Cas9 cleavage with a 1-bp insertion (T) at one end, and a 2-bp insertion (GT) at the opposite end (Fig. 5b).

### Growth and lipid production in genome-edited mutants

The volumetric biomass yields and lipid contents of the knockout strains of the *CDMT1*, *DMAN1*, and *AATPL1* genes were examined by cultivating them in 1/5 strength urea-phosphate (1/5 UP) medium for 14 days under continuous light.

The growth yields of the two *CDMT1*-knockout strains, CR24 and CR26, determined as cell-mass dry weight per liter of culture, were nearly equal to the wild-type strain, whereas that of strain PK4 was significantly lower than those of the other strains (Fig. 3c). The lipid contents of the *CDMT1*-knockout strains at days 7, 11, and 14 were significantly lower than that of strain PK4, and almost equal to the wild-type strain (Fig. 3d). From these results, it was concluded that the *CDMT1* mutation was not the cause of higher lipid accumulation in strain PK4.

The growth yields of the two knockout strains of *DMAN1*, namely strains CR189 and CR193, at days 11 and 14 were significantly lower than that of the wild-type strain and slightly higher than that of strain PK4 (Fig. 4c). The lipid contents in the two knockout strains at days 7, 11, and 14 were significantly lower than that of strain PK4 and almost the same as that of the wild-type strain (Fig. 4d). These results indicated that the mutation in *DMAN1* was not the cause of the high-lipid phenotype of strain PK4 but one of the causes of the low growth yield of strain PK4.

**Fig. 6:**
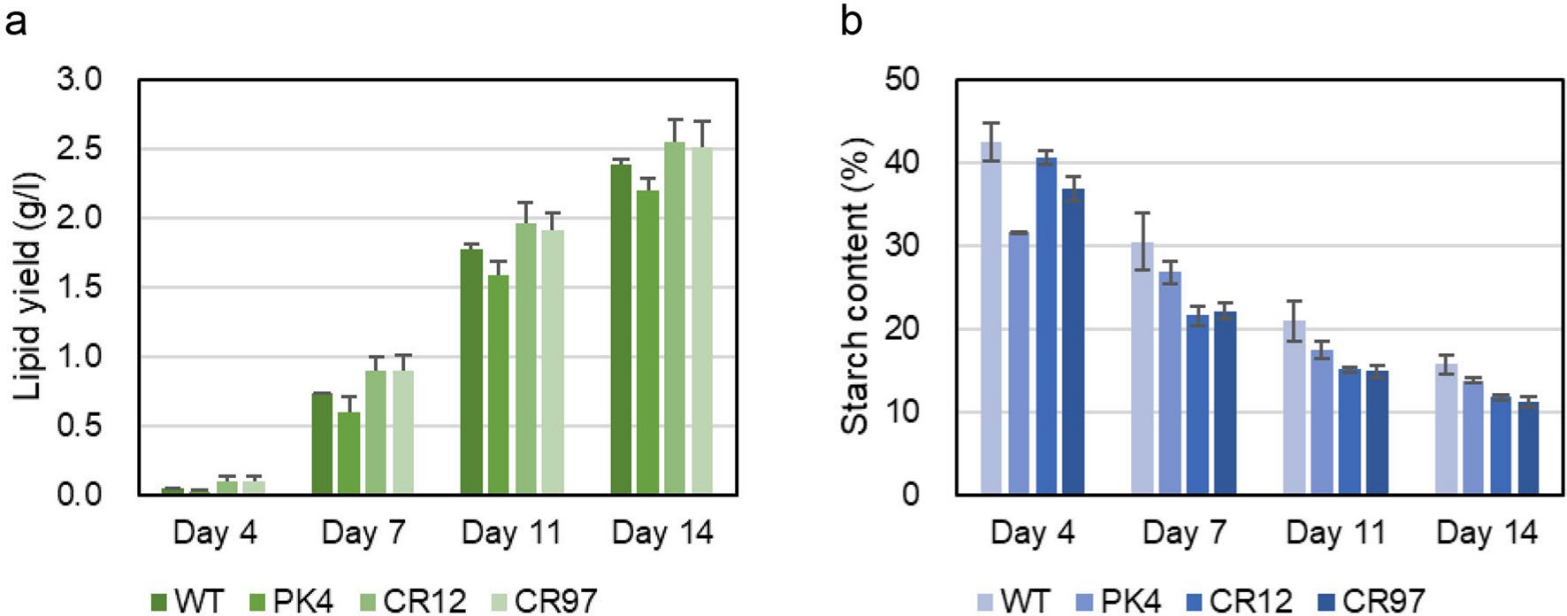
Volumetric lipid yield and starch content of the wild-type strain, strain PK4, and *AATPL1*-knockout strains under continuous light conditions. **a** Volumetric lipid yield [volumetric biomass yield × lipid content]. **b** Starch content in percentage of dry weight biomass (w/w). Bars represent standard deviations of the data from three independent samples. Statistical significance of differences between the values of strain PK4 and those of other strains or between the values of the wild-type strain and those of other strains were tested by Student’s t test (two-tailed), and the results are shown in Additional file 7: Table S3

The lipid contents in the two *AATPL1*-knockout strains, strains CR12 and CR97, at days 11 and 14 were almost the same as that of strain PK4 and significantly higher than that of the wild-type strain (Fig. 5d). This result indicates that the mutation in *AATPL1* was the cause of the high-lipid phenotype observed in strain PK4. On the other hand, the growth yields of two knockout strains were similar to that of the wild-type strain but significantly higher than that of strain PK4 (Fig. 5c). Thus, two phenotypes of strain PK4, namely higher lipid content and lower growth yield than those of the wild-type strain, were caused by separate mutations. The volumetric lipid yield of the wild-type strain or its derivatives was calculated by multiplying the volumetric biomass yield by lipid content. Those of the *AATPL1*-knockout strains were significantly higher than those of strain PK4 and slightly higher than that of the wild-type strain (Fig. 6a). The starch contents of strain PK4 and the *AATPL1*-knockout strains (CR12 and CR97) were lower than that of the wild-type strain (Fig. 6b).

### Growth and lipid production in the *AATPL1*-knockout mutants under diurnal rhythms

*A. thaliana* possesses two genes for AATPs, At1g80300 and At1g15500, encoding AATP1 and AATP2, respectively. The loss of function of AATP1 marginally affects plant development. Similarly, mutants of *A. thaliana* defective in *AAT2* grew normally under long-day conditions. However, under short-day conditions with low light, their growth is severely impaired [39]. Thus, in *A. thaliana*, nocturnal ATP import into the plastid seems to be required for proper anabolic metabolism and normal plant development.

**Fig. 7:**
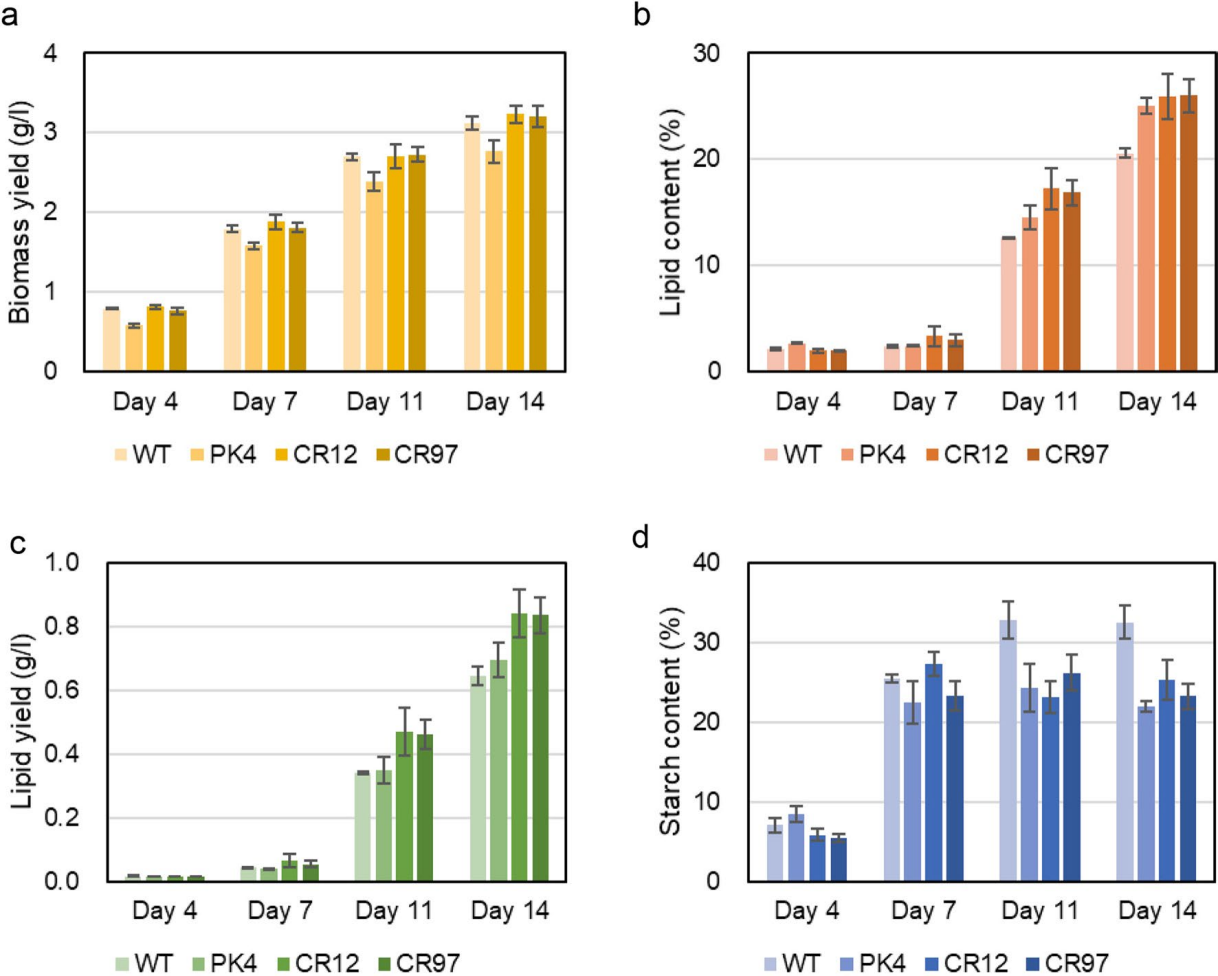
Growth and contents of lipid and starch in the wild-type strain, strain PK4, and *AATPL1*-knockout strains under the L/D 10:14 cycle. **a** Volumetric biomass yield [cell mass dry weight per liter of culture (g l^−1^)]. **b** Lipid content in percentage of dry weight biomass (w/w). **c** Volumetric lipid yield (g l^−1^). **d** Starch content in percentage of dry weight biomass (w/w). Bars represent standard deviations of the data from three independent samples. Statistical significance of differences between the values of strain PK4 and those of other strains or between the values of the wild-type strain and those of other strains were tested by Student’s t test (two-tailed), and the results are shown in Additional file 7: Table S4

**Fig. 8:**
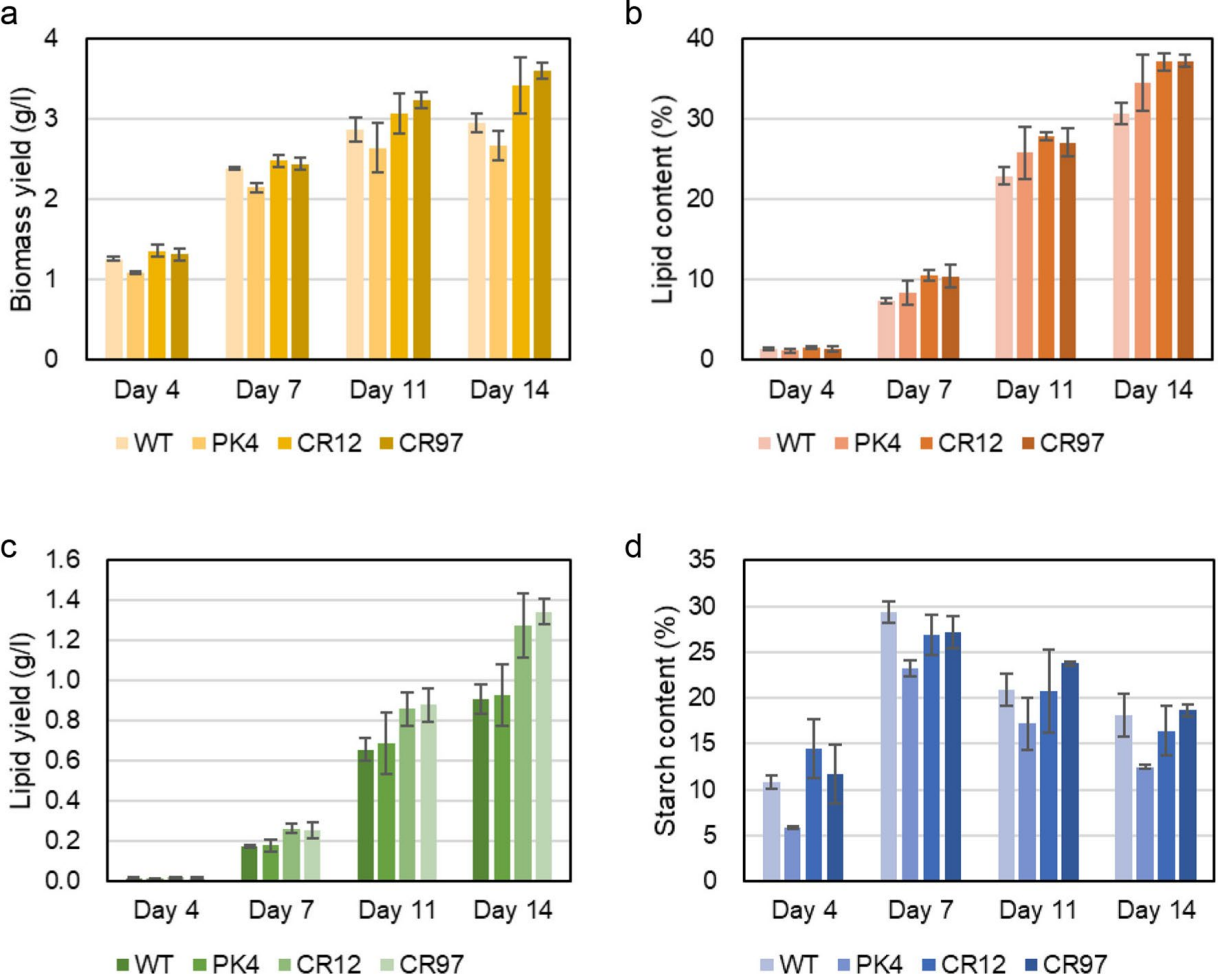
Growth and the contents of lipid and starch in the wild-type strain, strain PK4, and *AATPL1*-knockout strains under the L/D 16:8 cycle. **a** Volumetric biomass yield [cell mass dry weight per liter of culture (g l^−1^)]. **b** Lipid content in percentage of dry weight biomass (w/w). **c** Volumetric lipid yield (g l^−1^). **d** Starch content in percentage of dry weight biomass (w/w). Bars represent standard deviations of the data from three independent samples. Statistical significance of differences between the values of strain PK4 and those of other strains or between the values of the wild-type strain and those of other strains were tested by Student’s t test (two-tailed), and the results are shown in Additional file 7: Table S5

**Fig. 9:**
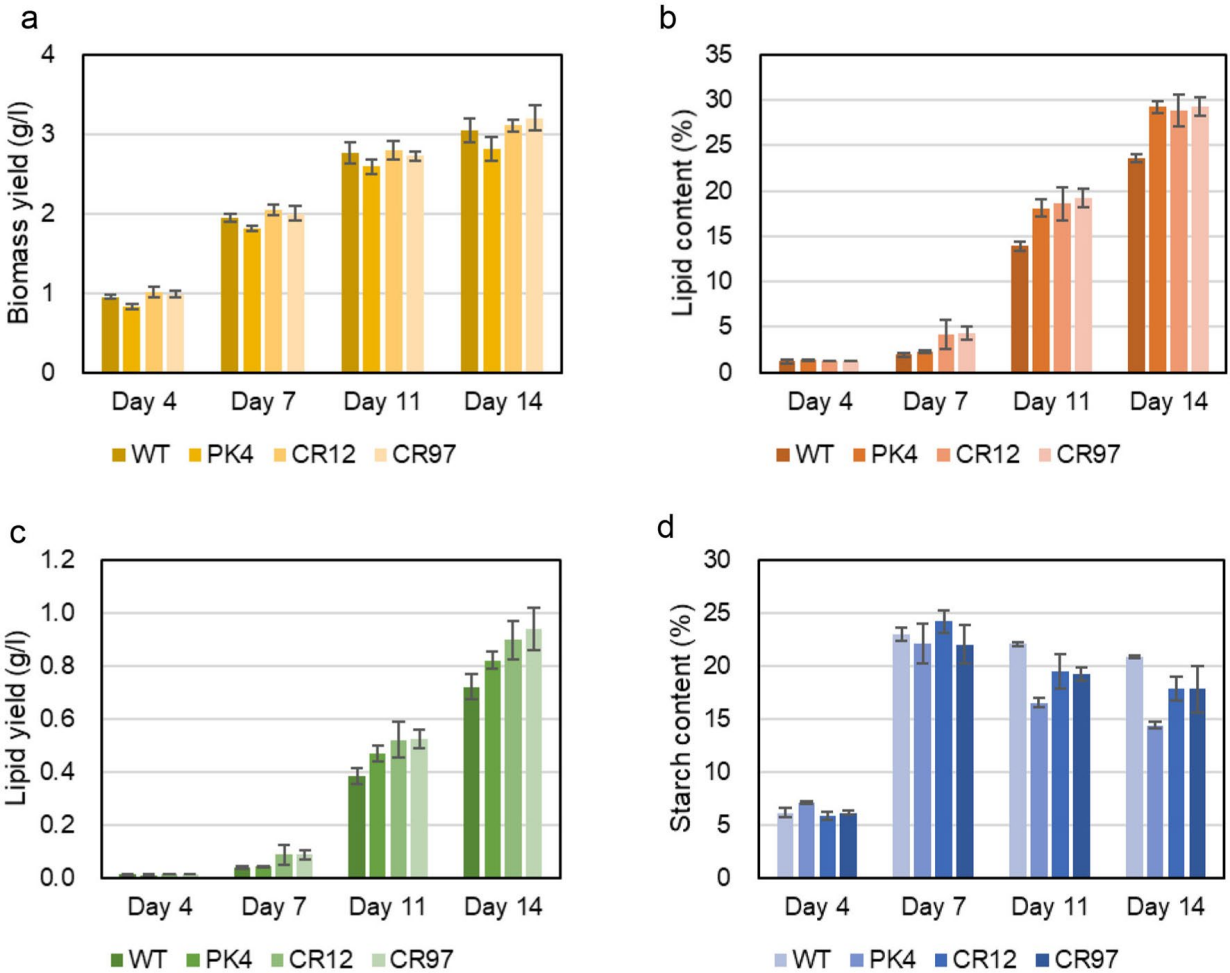
Growth and the contents of lipid and starch in the wild-type strain, strain PK4 and *AATPL1*-knockout strains under the L/D 12:12 cycle. **a** Volumetric biomass yield [cell mass dry weight per liter of culture (g l^−1^)]. **b** Lipid content in percentage of dry weight biomass (w/w). **c** Volumetric lipid yield (g l^−1^). **d** Starch content in percentage of dry weight biomass (w/w). Bars represent standard deviations of the data from three independent samples. Statistical significance of differences between the values of strain PK4 and those of other strains or between the wild-type strain and those of other strains were tested by Student’s t test (two-tailed), and the results are shown in Additional file 7: Table S6

Considering the results of the *A. thaliana AAT2* mutants, strain NIES-2152, strain PK4, and the two *AATPL1*-knockout mutants (strains CR12 and CR97) were grown under different L/D cycles, namely 16:8 h-, 12:12 h-, and 10:14 h cycles. By reducing the day length from 24 to 10 h, the growth yields of all strains at day 14 decreased significantly: from 4.9 to 3 g l^−1^for strain NIES-2152, from 4.2 to 2.8 g l^−1^ for strain PK4, and from 4.9 to 3.2 g l^−1^ for the two *AATPL1*-knockout strains (Fig. 7a). The growth yields of each strain were comparable under the three different L/D cycles (Figs. 8, 9). These results revealed that the phenotypic changes of the *AATPL1* mutants under short-day conditions were less severe than those of the *A. thaliana AAT2* mutants.

The lipid contents per cell dry weight of strain NIES-2152 and its derivatives defective in *AATPL1*, strains PK4, CR12, and CR97, at day 14 were almost 50% (w/w) or more when they were grown under continuous light. However, their lipid contents decreased as the day length decreased. Under all light conditions examined, the lipid contents of strains PK4, CR12, and CR97 were always higher than that in the wild-type strain. Lipid productivity (g l^−1^ day^−1^) is an important index for economical feasibility of biodiesel production. In *AATPL1*-knockout strains, these values were 0.23 ± 0.01, 0.15 ± 0.01, 0.12 ± 0.01, and 0.11 ± 0.01 under day lengths of 24, 16, 12, and 8 h, respectively, which were 3.3%, 43%, 22%, and 29% higher than those of the wild-type strain. Thus, the *AATPL1*-knockout strains may be promising candidates for outdoor production of biofuels.

## Conclusions

In this study, we established a CRISPR/Cas9-mediated gene-editing method for *P. kessleri* and created mutant strains that exhibited significantly higher lipid productivity than the wild-type strain under diurnal rhythms. However, the gene knock-in frequency with this procedure was low, ranging from 1.5 to 7.2 × 10^−7^ per input cell. Moreover, there is public concern about horizontal transfer of antibiotic resistance genes from GM microalgae grown in massive outdoor cultures to other organisms. Therefore, we are currently focusing on the development of methods for the easy detection of CRISPR/Cas9-induced substitution and small-indel mutations, and for marker-free gene-editing.

## Methods

### Algal strains and culture conditions

The *P. kessleri* strain NIES-2152 was obtained from the Microbial Culture Collection at the National Institute for Environmental Studies (NIES) in Tsukuba, Japan. Strain NIES-2152 and its derivatives were cultured in 120-ml test tubes containing 50 ml BG-11 medium [40] under continuous illumination provided by daylight fluorescent tubes (40W FL40S • FR • P, Panasonic, Japan) at an intensity of 100 μmol m^−2^ s^−1^ in a plant-growth chamber (type #CLE‐303, TOMY, Japan). To induce lipid biosynthesis under nitrogen-depleted conditions, cells were grown in 1/5 UP medium [41]. These cultures were bubbled with 1% (v/v) CO_2_ at 25 °C. For agar plate preparation, media were solidified with 1.5% (w/v) agar (Bacto Agar, BD Difco, USA). The plates were then inoculated with strain NIES-2152 or its derivatives and incubated in a plant-growth chamber. When necessary, Zeocin^®^ at 35 μg ml^−1^ was included in the agar plates.

### Cell size measurement

A 1-ml cell suspension was sampled from cultures of strain NIES-2152 grown in BG-11 medium and centrifuged at 2000 g for 5 min at room temperature. Subsequently, cells in the pellet were fixed in 1 ml of BG-11 medium containing 1% (v/v) formaldehyde. The fixed cells were observed by light microscopy (BX51; Olympus, Japan). The microscopic images were recorded with a CCD camera, and cell sizes were determined using the ImageJ software [42].

### Construction of plasmid

For the isolation of genomic DNA, cells of strain NIES-2152 in 50 ml of culture were collected in a 1.5-ml screw-cap microtube (WATSON #1392–200, Japan) and frozen in liquid nitrogen. The frozen cells were then disrupted using pre-chilled metal crusher (TAITEC Corporation, Japan). The disrupted cells were suspended in TE buffer, and DNA was isolated from the lysate with phenol/chloroform/isoamyl alcohol extraction followed by ethanol precipitation. The DNA fraction was further purified with RNase A treatment, a second phenol/chloroform/isoamyl alcohol extraction, and a second ethanol precipitation.

PCRs were carried out in 50 μl of PCR buffer (Takara, Japan) containing an appropriate amount of DNA template, 10 pmol of the forward and reverse primers listed in Additional file 7: Table S1, and 25 μl of PrimeSTAR Max DNA polymerase Premix (2×), according to the manufacturer's protocols (Takara, Japan). PCR products were purified with NucleoSpin gel and PCR clean-up Kit (Takara, Japan).

The pbleHH plasmid (DDBJ/EMBL/GenBank accession number LC775354), carrying an expression construct of *ble* consisting of the promoter and terminator sequences of *HSP90* that frank the *ble* coding sequence, was constructed as follows. First, the 0.4-kb fragment comprising the *ble* coding sequence was PCR-amplified using the Hsp_ble_F- and Hsp_ble_R primers and 10 ng of pble-PeEGFP-KE1E DNA [27] as a template. In parallel, the 0.5-kb fragment comprising the *HSP90* promoter was PCR-amplified using 0.1 μg of the genomic DNA of strain NIES-2152 as a template and the HSP90P_F- and HSP90P_R primers; similarly, the 1.1-kb fragment comprising the *HSP90* terminator was PCR-amplified using 0.1 μg of the genomic DNA of strain NIES-2152 as a template and the HSP90T_F- and HSP90T_R primers. These three DNA fragments were assembled into a single fragment by PCR using 10 ng each of the three DNA fragments as templates and the HSP90P_F- and HSP90T_R primers. The resulting 1.7-kb DNA fragment was purified using the NucleoSpin Gel and PCR clean-up kit (Takara, Japan), digested using EcoRV and EcoRI, and cloned into the EcoRV and EcoRI sites of pBluescript II sk (+).

The pbleAA plasmid (DDBJ/EMBL/GenBank accession number LC775355), carrying the *ble* expression construct consisting of the promoter and the terminator sequences of *AATP1* that flank the *ble* coding sequence, was constructed as follows. The 0.4-kb fragment comprising the *ble* coding sequence was PCR-amplified using 10 ng of the pbleHH DNA as a template and ble_F- and ble_R primers. The 0.4-kb fragment comprising the promoter sequence of *AATP1* was PCR-amplified using 0.1 μg of the genomic DNA of strain NIES-2152 as a template and the AATP1P_F- and AATP1P_R primers. The 0.8-kb fragment comprising the terminator sequences of *AATP1* was PCR-amplified using 0.1 μg of the genomic DNA of strain NIES-2152 as a template and the AATP1T_F- and AATP1T_R primers. These three DNA fragments were assembled into a single DNA fragment by PCR using 10 ng each of the three fragments as templates and the AATP1P_F- and AATP1T_R primers. The resulting 1.6-kb DNA fragment was purified using the NucleoSpin Gel and PCR clean-up kit and cloned into the HincII site of pUC118.

The pbleRR plasmid (DDBJ/EMBL/GenBank accession number LC775356), carrying the *ble* expression construct consisting of the promoter and terminator sequences of *RBCS4* that flank the *ble* coding sequence, was constructed as follows. The 0.4-kb fragment of the *ble* coding sequence was PCR-amplified using 10 ng of pbleHH DNA as a template and the ble_F- and ble_R primers. The 0.9-kb DNA fragment comprising the promoter sequence of *RBCS4* was PCR-amplified using 0.1 μg of the genomic DNA of strain NIES-2152 as a template and the RBCS4P_F- and RBCS4P_R primers. The 0.9-kb DNA fragment comprising the terminator sequence of *RBCS4* was PCR-amplified using 0.1 μg of the genomic DNA of strain NIES-2152 as a template and the RBCS4T_F- and RBCS4T_R primers. These three DNA fragments were assembled into a single fragment by PCR using 10 ng each of the three fragments as templates and the RBCS4P_F- and RBCS4T_R primers. The resulting 2.1-kb DNA fragment was purified using the NucleoSpin Gel and PCR clean-up kit, and cloned into the HincII site of pUC118.

The pneoHH plasmid, carrying an expression construct of *neo* (neoHH) consisting of the promoter and terminator sequences of *HSP90* that frank the *neo* coding sequence, was constructed as follows. First, the 0.8-kb fragment comprising the *neo* coding sequence was PCR-amplified using the neo_F- and neo_R primers and 10 ng of pG418T1A DNA [30] as a template. The 4.2-kb DNA fragment comprising *HSP90* terminator sequence, pBluescript II sk ( +) plasmid backbone, and *HSP90* promoter sequence was PCR-amplified using 10 ng of pbleHH DNA as a template and the HSP90P_F2- and HSP90T_R2 primers. The two fragments were assembled into circular DNA using an In-Fusion cloning kit (Clontech, USA) according to the manufacturer’s instructions. The codons of *neo* were optimized based on the codon table of *P. kessleri* stored in the codon usage database at Kazusa DNA Research Institute (http://www.kazusa.or.jp/codon/). The optimized gene (*Pkneo*) was synthesized at FASMAC (Japan) and cloned into a pUCFa vector to construct pPkneo. The 0.8-kb fragment comprising the *Pkneo* coding sequence was PCR-amplified using the Pkneo_F- and Pkneo_R primers and 10 ng of pPkneo DNA as a template, while the 4.2-kb DNA fragment comprising (the *HSP90* terminator sequence)–(the backbone sequence of the pBluescript II sk ( +) plasmid)–(the *HSP90* promoter sequence), was PCR-amplified using 10 ng of pneoHH DNA as a template and the HSP90P_F3- and HSP90T_R3 primers. The two fragments were assembled into circular DNA using an In-Fusion cloning kit (Clontech, USA) to create the pPkneoHH plasmid.

The nucleotide sequences of all plasmids (Additional file 4: Figure S4) constructed were verified by Sanger sequencing on both strands. For genetic transformation, a DNA fragment containing a *ble*, *neo*, or *Pkneo* expression construct was prepared by PCR-amplification using 10 ng of each plasmid DNA as a template and an appropriate PCR-primer set shown in Additional file 7: Table S1. The PCR products were purified with the NucleoSpin Gel and PCR clean-up kit and used for genetic transformation.

### Delivery of RNP and/or DNA in cells of strain NIES-2152 using electroporation

Cells were precultured under an L/D 16:8 cycle at a light intensity of 100 µmol m^−2^ s^−1^ in BG-11 medium for 3–4 days until the cell density reached OD_750_ of 1.0. The preculture was diluted to OD_750_ of 0.0001–0.00005 in 50 ml of fresh BG-11 medium, and cells were grown under the same conditions as those in the preculture. On the fifth day, 2 h after the start of a light period, cells were harvested by centrifugation at 2900 g for 5 min, washed twice with 5 mM 2-morpholinoethanesulfonic acid buffer (pH 5.5), and suspended in Max Efficiency® Transformation Reagent for Alga (Thermo Fisher Scientific, USA) supplemented with 1% (w/v) glucose to cell densities of 1.0 × 10^9^ cells ml^−1^. The cell suspension was kept on ice prior to electroporation.

To deliver only DNA, 1 μg of a DNA fragment carrying a *ble*, neo, or *Pkneo* expression construct (bleHH, bleAA, bleRR, neoHH, and PkneoHH) was added to 30 μl of the cell suspension kept on ice, and the mixture was transferred to a 2-mm-gapped electroporation cuvette (EC-002S, Nepa Gene, Japan) to incubate at 16 ºC for 2 min. The cuvette was placed into an electroporator (ELEPO21, Nepa Gene, Japan), and its electrode impedance was checked to be higher than 4 kΩ. A single poring pulse (Pp) was applied at 1500–2500 V cm^−1^ with a pulse duration of 2.5–15 ms and a pulse interval of 50 ms. Immediately after the poring pulses, five transfer pulses (Tps) of alternative polarities (+ and −) were applied at 100–500 V cm^−1^ with a 50 ms pulse duration and a 50 ms pulse interval.

CRISPR/Cas9-mediated genome editing was performed as described previously [27] with some modifications. Since the genome editing frequency in *Coccomyxa*, belonging to the same class as strain NIES-2152 (Trebouxiophyceae), was very low, below 10^–4^ per input cell [27], bleHH was co-delivered with Cas9-RNP, and gene-edited clones were searched among Zeo^r^ transformants. CRISPR RNAs (crRNAs) were designed using the CRISPRdirect software [43], and listed in Additional file 7: Table S2. Potential off-target sites for each of the designed crRNAs were searched in the genome of strain NIES-2152 for 12-bp-long sequences adjacent to the PAM sequence that perfectly matches with the 3′-end sequence of the designed crRNAs. If the number of potential off-target sites for a crRNA was larger than one, this crRNA was not used for gene editing experiments. crRNAs and trans-activating crRNA (tracrRNA) were chemically synthesized by FASMAC (Japan). Three microliter of crRNA (200 pmol μl^−1^) and 3 μl of tracrRNA (200 pmol μl^−1^) were mixed in a screw-cap microtube and incubated at 95 °C for 5 min followed by slow cooling to hybridize crRNA and tracrRNA. Ten microliter of Cas9 nuclease (15 μg μl^−1^) (Nippon Gene, Japan) was diluted 50-fold with RNase-free water, then the volume of the protein solution was reduced below 10 μl with an Amicon Ultra spin filter (100 kDa, Milipore, USA), into which RNA-free water was added to the final volume of 60 μl. Thus, the total salt concentrations of the Cas9 nuclease solution were below 1 mM at which arcing during electroporation was not observed. Two microliter of hybridized crRNA-tracrRNA (200 pmol each) and 4 μl of Cas9 protein (10 μg) were mixed and incubated for at least 30 min to form RNP complexes. The RNP complexes thus formed were delivered into cells of strain NIES-2152 as follows. Thirty microliter of cell suspension containing 3 × 10^7^ cells prepared as described above, 6 μl of the RNP solution, and 1 μg of bleHH were mixed in a 2-mm gap electroporation cuvette, and electroporation was done as described above, except that a single Pp at 2500 V cm^−1^ with a 15-ms pulse duration followed by five Tp of alternative polarity at 250 V cm^−1^ with a 50-ms pulse duration and 50 ms pulse interval were applied.

After electroporation, cells were incubated on ice for 10 min, transferred in 1 ml of BG-11 medium containing 55 mM glucose, and incubated under dim light for recovery. In the case when only DNA of a *ble* expression construct was electroporated, cells were incubated at 25 °C for 24 h with gentle shaking under dim light. When both an RNP and DNA of a *ble* expression construct were electroporated, cells were incubated for 6 h at 37 °C, a temperature at which Cas9 nuclease shows optimum nuclease activity [44]. The incubation temperature was lowered to 25 °C, and the incubation was continued for a further 18 h with shaking. The 3 × 10^7^ cells were then spread on a BG-11 agar plate containing 35 μg ml^−1^ of Zeocin^Ⓡ^ and incubated in an incubator containing 1% (v/v) CO_2_ at 25 °C under continuous light at 100 μmol m^−2^ s^−1^.

Several single colonies grown on the plates were randomly selected for PCR analyses to detect the *ble* coding sequence. The templates for such PCR were prepared as follows: cells were picked up from each single colony grown on the plates, suspended in 50 μl of TE buffer containing 6% (w/v) Chelex-100 (BioRad, USA), and disrupted by boiling for 10 min. Cell debris was removed by centrifugation, and PCR was performed using the supernatant as a template. For the detection of the *ble* coding sequence, the ble_F- and ble_R primers (Additional file 7: Table S1) were used. For the detection of the *ble* coding sequence inserted at CRISPR/Cas9 target sites, PCR was performed using the primer sets that amplify DNA fragments encompassing CRISPR/Cas9 target sites (Additional file 7: Table S2). PCR products with sizes corresponding to the *ble*-inserted sequence were selected for Sanger sequencing to determine insertion loci.

### Measurement of lipid content

The lipid content of the lyophilized cells was determined using a benchtop, low-resolution pulsed NMR instrument (model MQC; Oxford Instruments, UK) following the International Standard Organization (ISO) 10565 protocol [45]. Olive oil was used as a standard [46–48]. Volumetric lipid yield (mg lipid l^−1^) was calculated by multiplying the volumetric biomass yield (mg dry weight of cells per liter of culture) by the lipid content.

### Measurement of starch content

Starch content in cells was determined using the reducing-sugar quantification method with dinitrosalicylic acid (DNS) reagent [49]. The DNS reagent was prepared by dissolving 5 g of DNS and 150 g of potassium sodium tartrate in 400 ml of 0.2 N NaOH at 80 °C, and the volume was adjusted to 500 ml with distilled water. A known dry weight of lyophilized cells was transferred to a screw-cap microtube (SSIbio #2330-00, USA) containing 1.0 ml of 80% ethanol and 1.0–1.5 g of Zilconia/Silica beads (ϕ = 0.1 mm, BioSpec, USA). The microtube was vigorously agitated using a bead beating grinder (FastPrep, MP Biomedicals, USA) set at a speed of 6.0 m s^−1^ for 1 min to homogenize the cells. The homogenized cells were then centrifuged to remove the supernatant containing soluble sugars, and the pellet was suspended in 1 ml of MilliQ water and 50 µl of 2 M sodium acetate. Heat-stable alpha-amylase (A3306, Sigma-Aldrich, USA) was added at an amount greater than 200 units to the solution, and the mixture was incubated at 80 °C for 1 h. After centrifugation, 100 µl of the supernatant was mixed with an equal volume of DNS reagent and heated at 100 °C for 20 min. Absorbance at 550 nm was then measured. The concentration of starch was calculated from a calibration curve prepared with known quantities of soluble starch (Nacalai Tesque, Japan) that underwent hydrolysis with the heat-stable amylase.

### Supplementary Information

**Additional file 1: Figure S1. **Structure of *CDMT1*, *DMAN1*, and *AATPL1*. a. Structure of *CDMT1*. Black boxes represent exons, thin lines represent introns, and red arrows represent target sites of crRNAs. The yellow bar underneath the gene indicates a conserved domain in this gene. b. Structure of the *DMAN1*. Black boxes represent exons, thin lines represent introns, and red arrows represent target sites of crRNAs. Two yellow bars underneath the gene indicate conserved domains in this gene. c. Structure of *AATPL1*. Black boxes represent exons, thin lines represent introns, and red arrows represent target sites of crRNAs. The yellow bars underneath the gene indicate conserved domains in this gene.

**Additional file 2: Figure S2.** Light microscopic images of cells of strain NIES-2152 under diurnal rhythms. Cells of strain NIES-2152 were cultured in BG-11 medium under the L/D 16:8 cycle. Samples were taken at different time points during the 3rd dark period and 4th light period.

**Additional file 3: Figure S3. **Antibiotics susceptibility tests for strain NIES-2152. a. NIES-2152 cells were suspended to a density of 1 × 10^8^ cells ml^−1^, and 100 μl of the cell suspension were spotted on BG-11 agar plates containing Zeocin® (0, 25, or 35 μg ml^−1^) or G418 (0, 15, or 25 μg ml^−1^). The plates were incubated at 25 °C under continuous light at 100 μmol m^−2^ s^−1^ for 14 days. The growth of NIES-2152 was inhibited at 35 μg ml^−1^ of Zeocin and at 25 μg ml^−1^ of G418, respectively. These concentrations were used for selection of transformants. b. bleHH was delivered in cells of strain NIES-2152 as described in the Methods section. The electroporated cells were spread on a BG-11 agar plate containing 35 μg ml^−1^ Zeocin®, and incubated at 25 °C under continuous illumination at 100 μmol m^−2^ s^−1^ in a plant-growth chamber for 14 days.

**Additional file 4: Figure S4.** Structures of plasmids used in this study. Abbreviations: *HSP90_P*: the promoter region of the gene encoding heat shock protein 90 (PkHSP90), *HSP90_T*: the terminator region of the gene encoding PkHSP90, *AATP1_P*: the promoter region of the gene encoding plastidic ATP/ADP translocase 1 (PkAATP1), *AATP1_T*: the terminator region of the gene for PkAATP1, *RBSC4_P*: the promoter region of the gene encoding ribulose bisphosphate carboxylase/oxygenase small subunit 4 (PkRBCS4), *RBCS4_T*: the terminator region of the gene for PkRBCS4, *ble*: the coding sequence of the *ble* gene conferring resistance to bleomycin/phleomycin/ Zeocin®, *neo*: the coding sequence of the *neo* gene conferring to neomycin/G418, and *Pkneo*: the codon-optimized *neo* gene.

**Additional file 5: Figure S5.** PCR detection of the *ble* DNA in the genomes of Zeo^r^ transformants of strain NIES-2152. Genomic DNAs were isolated from cells of strain NIES-2152 and Zeo^r^ transformants as described in the Methods section. A 375-bp-long partial sequence of *ble* integrated in the genomic DNAs was amplified by PCR with the primer set, ble_F and ble_R (Additional file 7: Table S1). PCR products of the expected size were amplified from all 116 genomic DNAs examined. M: a molecular size marker (100 bp DNA ladder, Takara, Japan). The templates used were: P: pbleHH plasmid DNA, W: genomic DNA of strain NIES-2152, N: no template, and 1–116: genomic DNAs of Zeo^r^ transformants of strain NIES-2152.

**Additional file 6: Figure S6.** PCR identification of *DMAN1*-knockin mutants. Zeocin®-resistant transformants were obtained after electroporation of a mixture of bleHH and one of three gRNA/Cas9 complexes comprising DMAN1_1-, DMAN1_2-, or DMAN1_3 crRNA. Genomic DNAs were isolated from cells of strain NIES-2152 and its Zeo^r^ transformants using the method described in the Methods section. A 1,492-bp-long region of *DMAN1* containing the three crRNA recognition sequences was PCR amplified from 114 Zeo^r^ transformants using the primer set, DMAN1_F and DMAN1_R (Additional file 7: Table S1). Among the PCR products obtained from 114 Zeo^r^ transformants, PCR products from 7 transformants obtained with each of the three different crRNAs are presented in this figure. M: a molecular size marker (λ-EcoT14 I digest). The templates used were: W: genomic DNA of strain NIES-2152, N: no template, and 160–226: genomic DNAs of Zeo^r^ transformants of strain NIES-2152. Arrows indicate PCR products from the bleHH knockin clones.

**Additional file 7: Table S1.** Primers used in this study. **Table S2.** crRNA sequences. **Table S3.** Statistical significance of the difference in growth- and lipid yields between the wild-type strain and other strains or between strain PK4 and other strains grown under continuous light conditions. **Table S4.** Statistical significance of the difference in growth- and lipid yields between wild-type and other strains or between strain PK4 and other strains grown under the L/D 10:14 cycle. **Table S5.** Statistical significance of the difference in growth- and lipid yields between wild-type and other strains or between strain PK4 and other strains grown under the L/D 16:8 cycle. **Table S6.** Statistical significance of the difference in growth- and lipid yields between wild-type and other strains or between strain PK4 and other strains grown under the L/D 12:12 cycle.

## Data Availability

All data generated or analyzed during this study are included in this published article and its supplementary information files.
