# Supplementary figures and images for: Development of a CRISPR/Cas9-mediated gene-editing method to isolate a mutant of the unicellular green alga Parachlorella kessleri strain NIES-2152 with improved lipid productivity
Source: Biotechnol Biofuels Bioprod. 2024 Mar 5;17:36. doi: 10.1186/s13068-024-02484-7 (PMC10916037; doi:10.1186/s13068-024-02484-7)

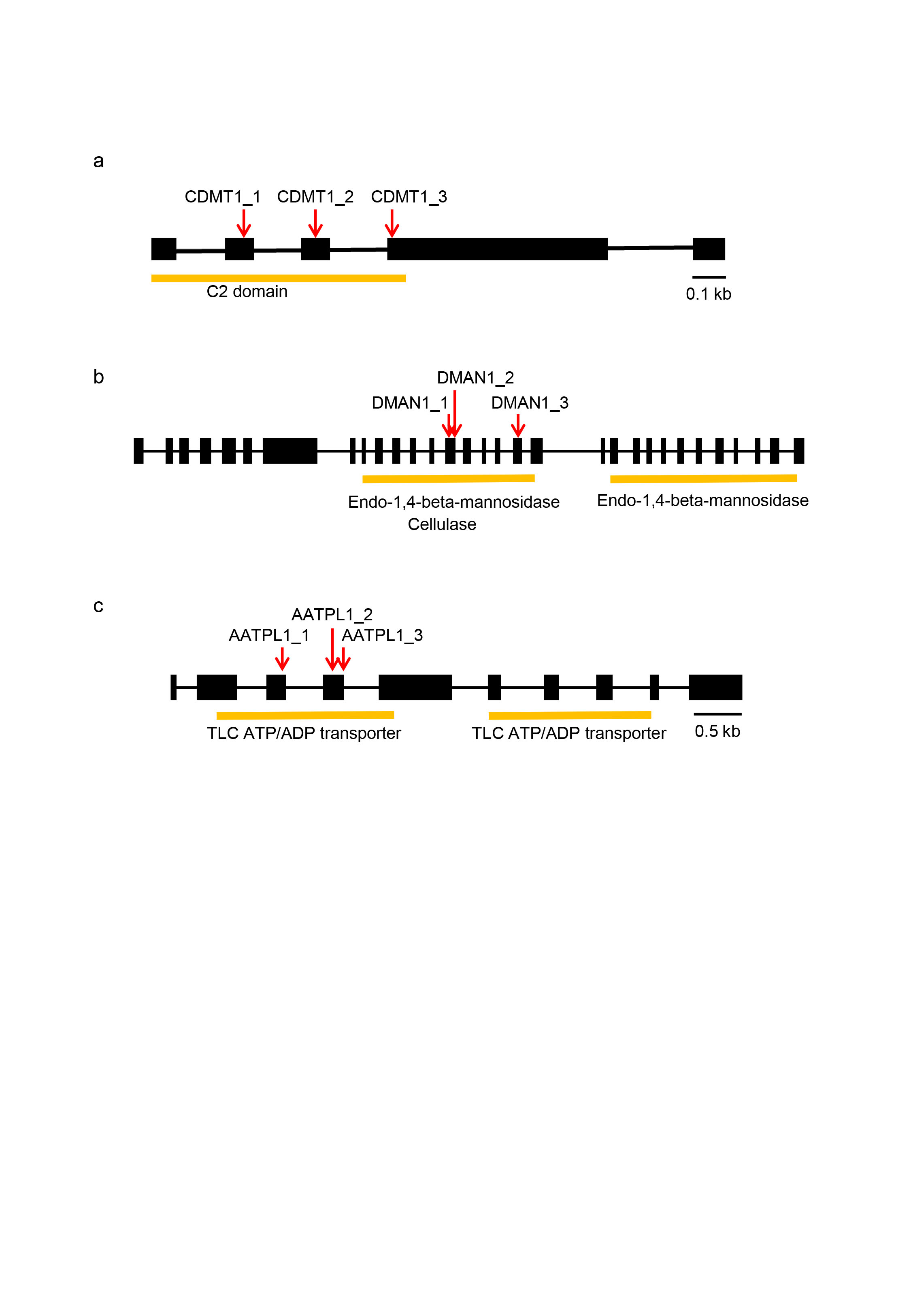

Supplement: Supplementary file 1 — Additional file 1: Figure S1. Structure of CDMT1, DMAN1, and AATPL1. a. Structure of CDMT1. Black boxes represent exons, thin lines represent introns, and red arrows represent target sites of crRNAs. The yellow bar underneath the gene indicates a conserved domain in this gene. b. Structure of the DMAN1. Black boxes represent exons, thin lines represent introns, and red arrows represent target sites of crRNAs. Two yellow bars underneath the gene indicate conserved domains in this gene. c. Structure of AATPL1. Black boxes represent exons, thin lines represent introns, and red arrows represent target sites of crRNAs. The yellow bars underneath the gene indicate conserved domains in this gene. [file 13068_2024_2484_MOESM1_ESM.jpg]

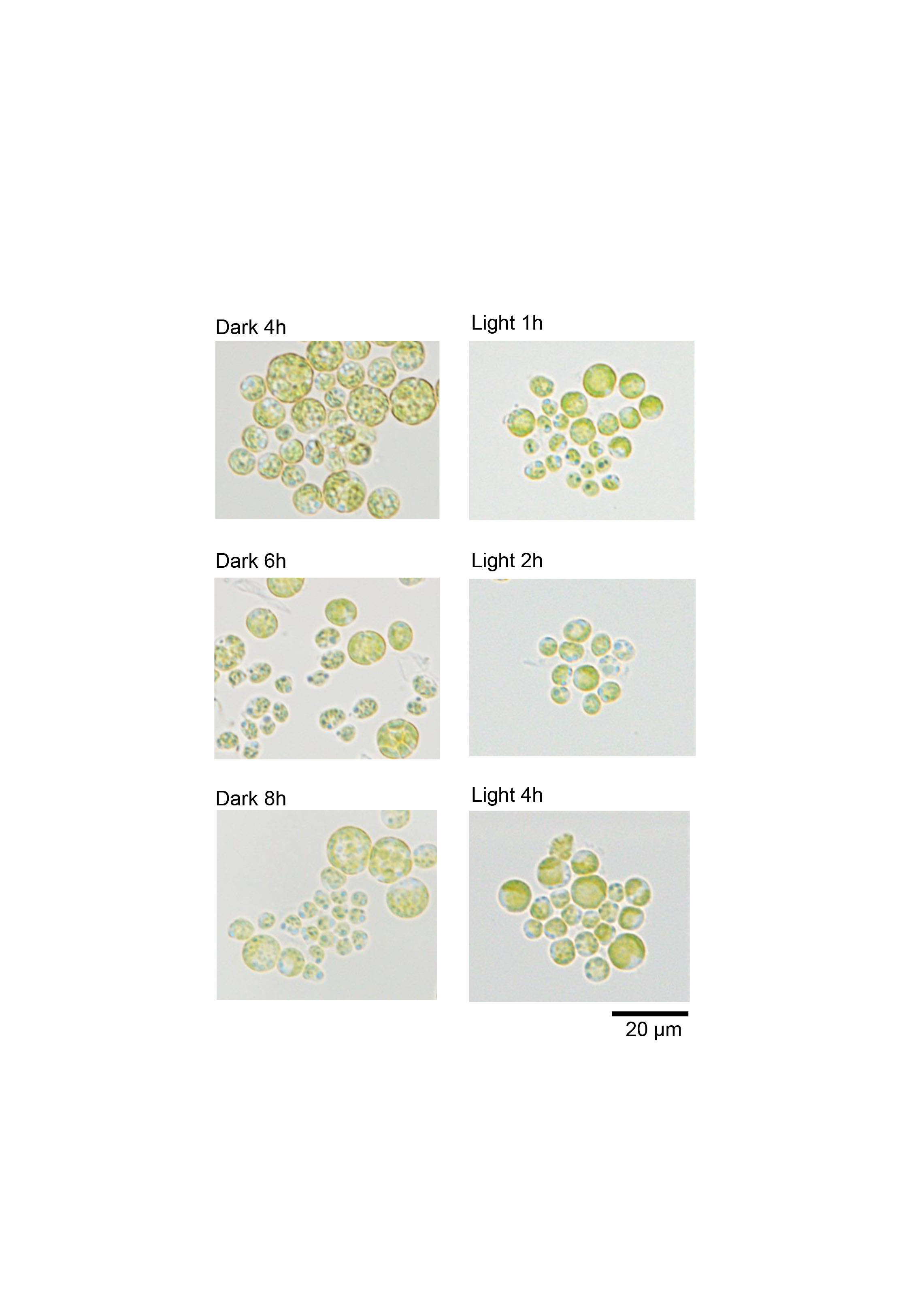

Supplement: Supplementary file 2 — Additional file 2: Figure S2. Light microscopic images of cells of strain NIES-2152 under diurnal rhythms. Cells of strain NIES-2152 were cultured in BG-11 medium under the L/D 16:8 cycle. Samples were taken at different time points during the 3rd dark period and 4th light period. [file 13068_2024_2484_MOESM2_ESM.jpg]

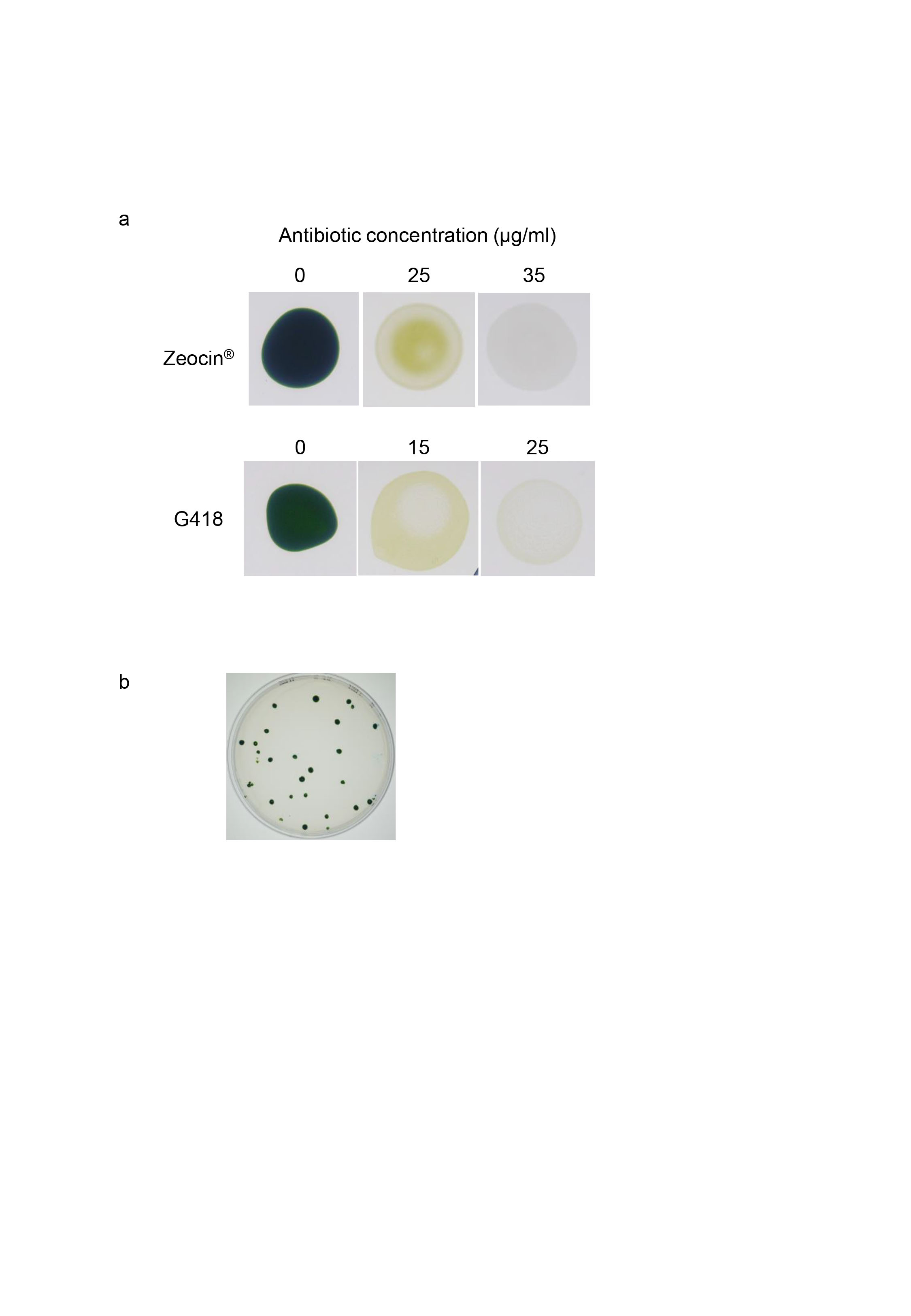

Supplement: Supplementary file 3 — Additional file 3: Figure S3. Antibiotics susceptibility tests for strain NIES-2152. a. NIES-2152 cells were suspended to a density of 1 × 10^8^ cells ml−1, and 100 μl of the cell suspension were spotted on BG-11 agar plates containing Zeocin® (0, 25, or 35 μg ml−1) or G418 (0, 15, or 25 μg ml−1). The plates were incubated at 25 °C under continuous light at 100 μmol m−2 s−1 for 14 days. The growth of NIES-2152 was inhibited at 35 μg ml−1 of Zeocin and at 25 μg ml−1 of G418, respectively. These concentrations were used for selection of transformants. b. bleHH was delivered in cells of strain NIES-2152 as described in the Methods section. The electroporated cells were spread on a BG-11 agar plate containing 35 μg ml−1 Zeocin®, and incubated at 25 °C under continuous illumination at 100 μmol m−2 s−1 in a plant-growth chamber for 14 days. [file 13068_2024_2484_MOESM3_ESM.jpg]

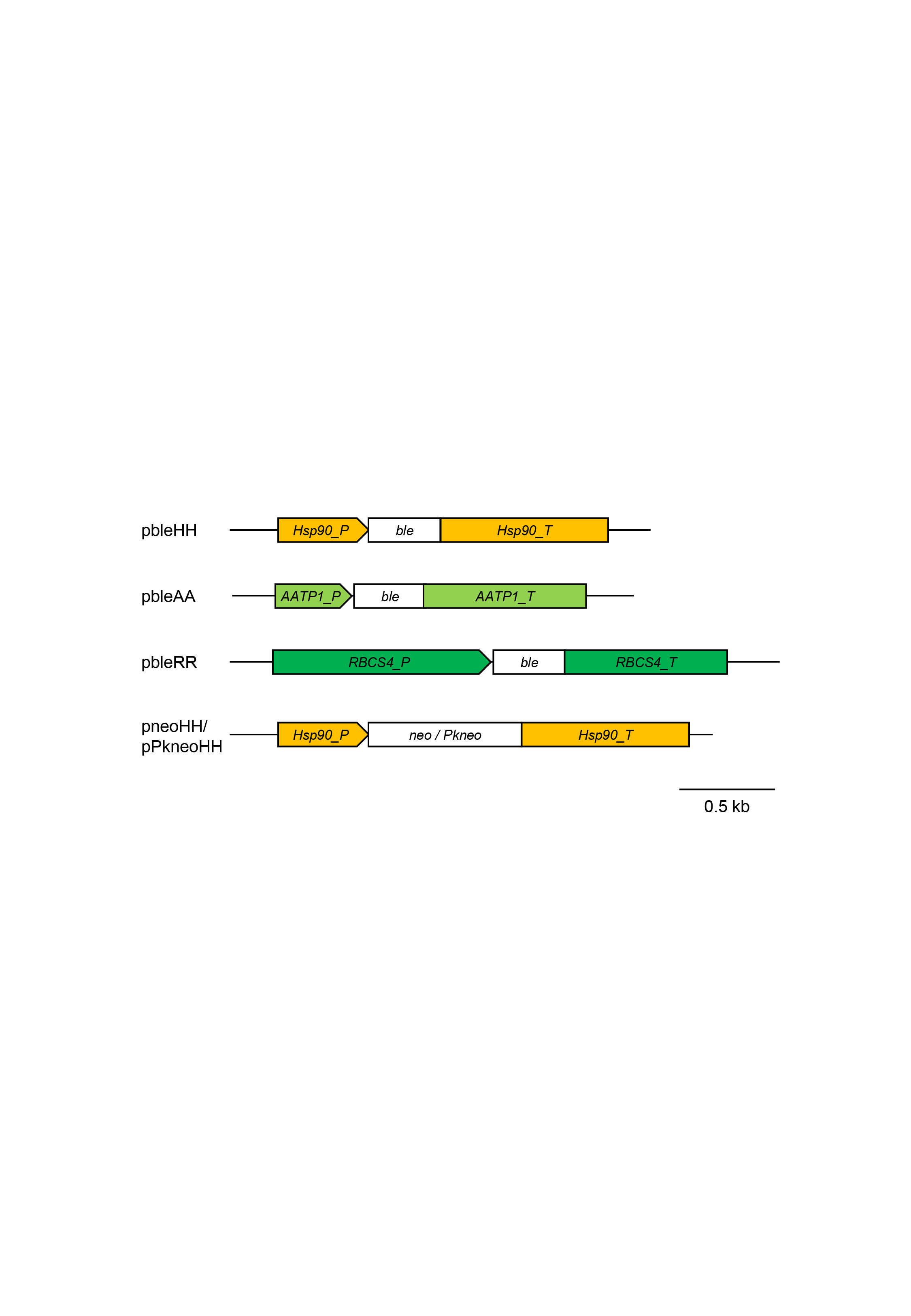

Supplement: Supplementary file 4 — Additional file 4: Figure S4. Structures of plasmids used in this study. Abbreviations: HSP90_P: the promoter region of the gene encoding heat shock protein 90 (PkHSP90), HSP90_T: the terminator region of the gene encoding PkHSP90, AATP1_P: the promoter region of the gene encoding plastidic ATP/ADP translocase 1 (PkAATP1), AATP1_T: the terminator region of the gene for PkAATP1, RBSC4_P: the promoter region of the gene encoding ribulose bisphosphate carboxylase/oxygenase small subunit 4 (PkRBCS4), RBCS4_T: the terminator region of the gene for PkRBCS4, ble: the coding sequence of the ble gene conferring resistance to bleomycin/phleomycin/ Zeocin®, neo: the coding sequence of the neo gene conferring to neomycin/G418, and Pkneo: the codon-optimized neo gene. [file 13068_2024_2484_MOESM4_ESM.jpg]

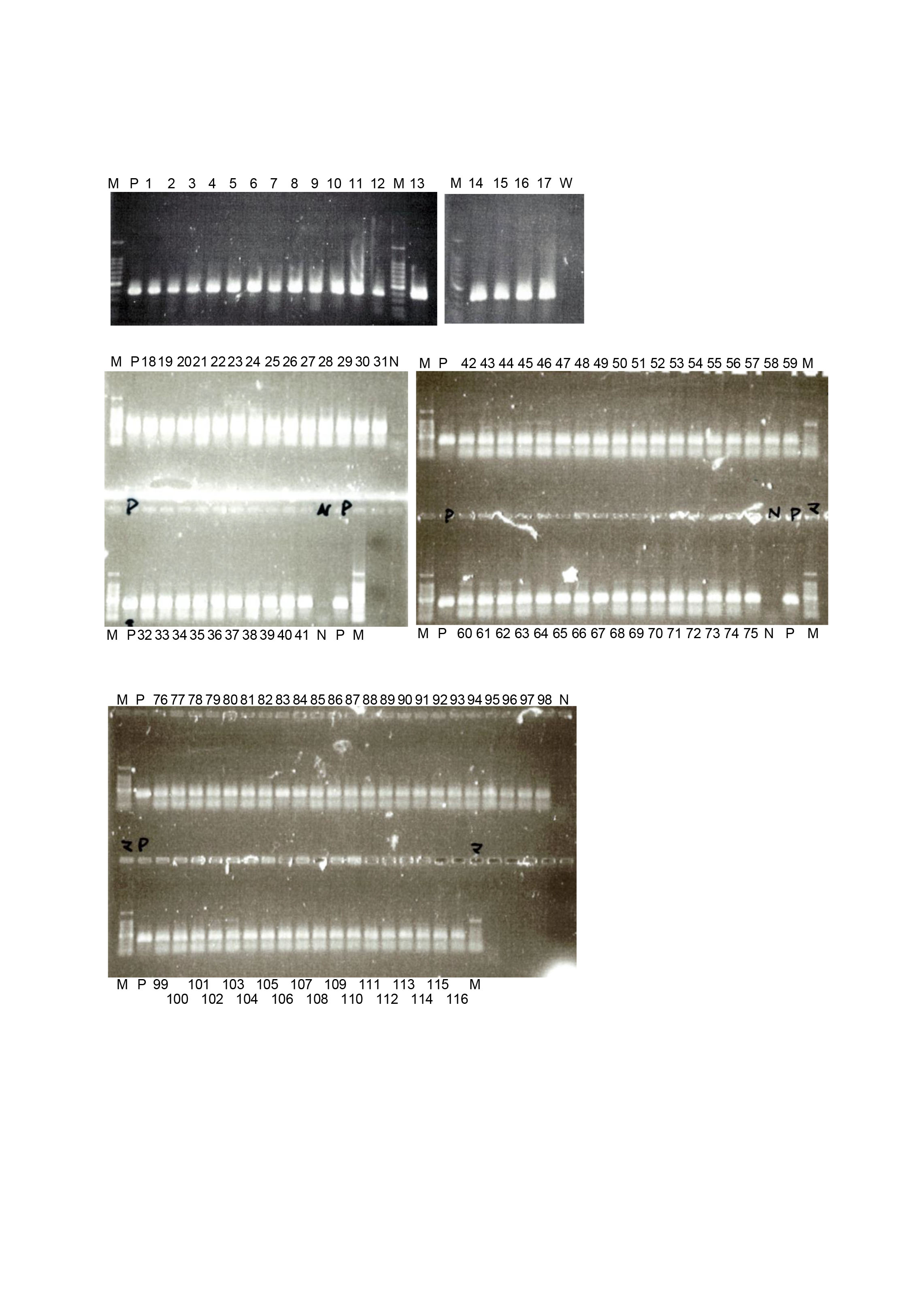

Supplement: Supplementary file 5 — Additional file 5: Figure S5. PCR detection of the ble DNA in the genomes of Zeor transformants of strain NIES-2152. Genomic DNAs were isolated from cells of strain NIES-2152 and Zeor transformants as described in the Methods section. A 375-bp-long partial sequence of ble integrated in the genomic DNAs was amplified by PCR with the primer set, ble_F and ble_R (Additional file 7: Table S1). PCR products of the expected size were amplified from all 116 genomic DNAs examined. M: a molecular size marker (100 bp DNA ladder, Takara, Japan). The templates used were: P: pbleHH plasmid DNA, W: genomic DNA of strain NIES-2152, N: no template, and 1–116: genomic DNAs of Zeor transformants of strain NIES-2152. [file 13068_2024_2484_MOESM5_ESM.jpg]

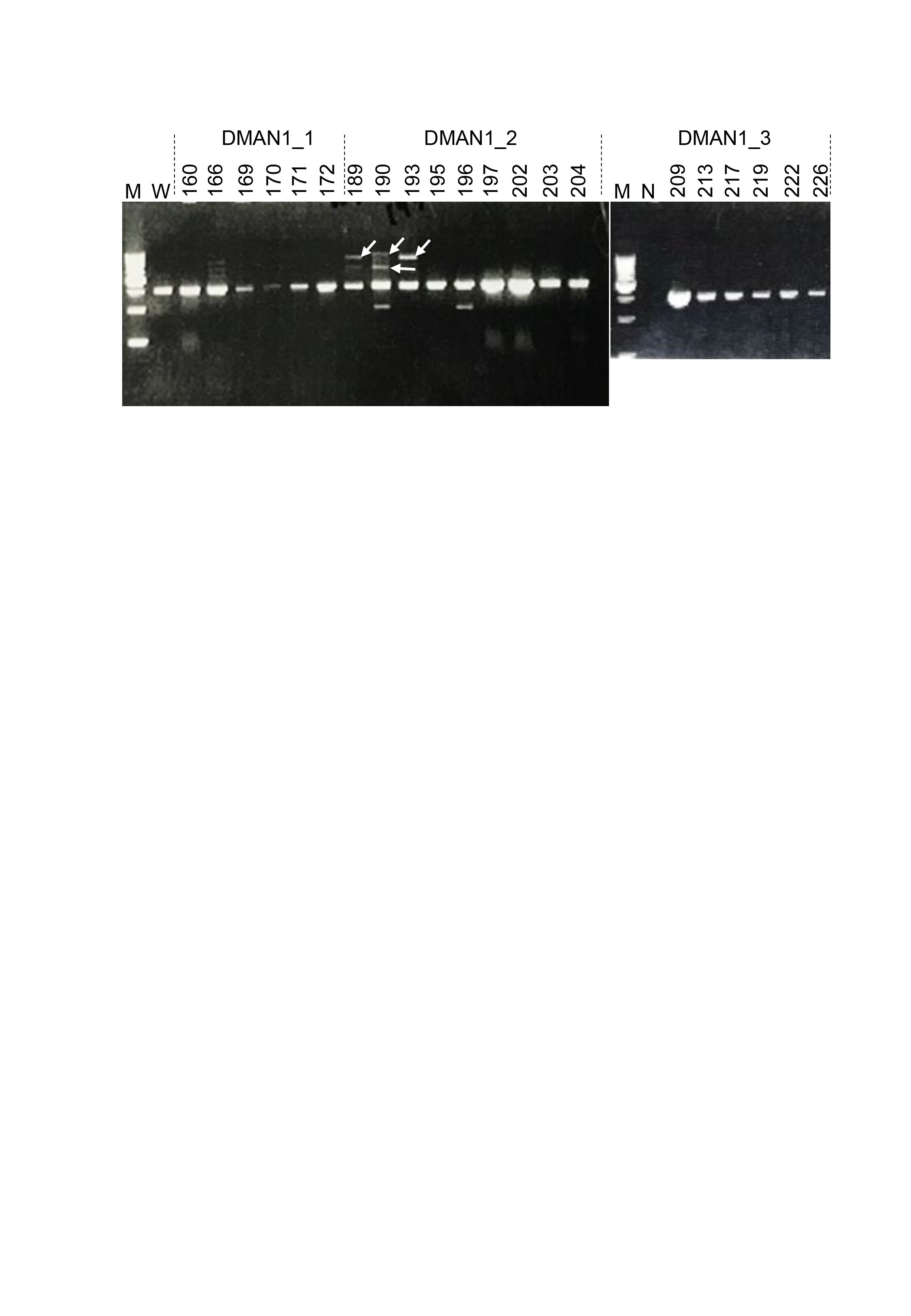

Supplement: Supplementary file 6 — Additional file 6: Figure S6. PCR identification of DMAN1-knockin mutants. Zeocin®-resistant transformants were obtained after electroporation of a mixture of bleHH and one of three gRNA/Cas9 complexes comprising DMAN1_1-, DMAN1_2-, or DMAN1_3 crRNA. Genomic DNAs were isolated from cells of strain NIES-2152 and its Zeor transformants using the method described in the Methods section. A 1,492-bp-long region of DMAN1 containing the three crRNA recognition sequences was PCR amplified from 114 Zeor transformants using the primer set, DMAN1_F and DMAN1_R (Additional file 7: Table S1). Among the PCR products obtained from 114 Zeor transformants, PCR products from 7 transformants obtained with each of the three different crRNAs are presented in this figure. M: a molecular size marker (λ-EcoT14 I digest). The templates used were: W: genomic DNA of strain NIES-2152, N: no template, and 160–226: genomic DNAs of Zeor transformants of strain NIES-2152. Arrows indicate PCR products from the bleHH knockin clones. [file 13068_2024_2484_MOESM6_ESM.jpg]
