# Supplementary material for: Development of a CRISPR/Cas9-mediated gene-editing method to isolate a mutant of the unicellular green alga Parachlorella kessleri strain NIES-2152 with improved lipid productivity
Source: Biotechnol Biofuels Bioprod. 2024 Mar 5;17:36. doi: 10.1186/s13068-024-02484-7 (PMC10916037; doi:10.1186/s13068-024-02484-7)
Supplement: Supplementary file 7 — Additional file 7: Table S1. Primers used in this study. Table S2. crRNA sequences. Table S3. Statistical significance of the difference in growth- and lipid yields between the wild-type strain and other strains or between strain PK4 and other strains grown under continuous light conditions. Table S4. Statistical significance of the difference in growth- and lipid yields between wild-type and other strains or between strain PK4 and other strains grown under the L/D 10:14 cycle. Table S5. Statistical significance of the difference in growth- and lipid yields between wild-type and other strains or between strain PK4 and other strains grown under the L/D 16:8 cycle. Table S6. Statistical significance of the difference in growth- and lipid yields between wild-type and other strains or between strain PK4 and other strains grown under the L/D 12:12 cycle. [file 13068_2024_2484_MOESM7_ESM.docx]

**Additional table 1**

**Primers used in this study**

| Primer | Sequence (5'to 3') |
| --- | --- |
| For plasmid construction | |
| HSP90P_F | GATAAGCTTGATATCCTTGGGCATCAACTGAGTTA |
| HSP90P_R | GCTTGGCCATTATGGCTTCCTGACCCTGAAG |
| HSP90P_R2 | AATGGCCGATCCCATTATGGCTTCCTGACCCTGA |
| HSP90P_R3 | AATAGCGCTGCCCATTATGGCTTCCTGACCCTGAAGCGCA |
| HSP90T_F | AGGACTGAGCAACTAGCATTAGAAAGGTGCCA |
| HSP90T_F2 | CGAGTTCTTCTGAGCAACTAGCATTAGAAAGGT |
| HSP90T_F3 | GACGAGTTCTTCTAAGCAACTAGCATTAGAAAGGTGCCA |
| HSP90T_R | GGGCTGCAGGAATTCGGTTGCTGTGCTGCCTTACTTGCT |
| Hsp_ble_F | AGCCATAATGGCCAAGCTGACCAGCGCC |
| Hsp_ble_R | TGCTAGTTGCTCAGTCCTGCTCCTCGGCCACGAAG |
| RBCS4P_F | TAGTGCTGCCGGAGCTTGCAACT |
| RBCS4P_R | GGTCAGCTTGGCCATAGTACCGATCGGAAGGTGCTAAG |
| RBCS4T_F | GAGGAGCAGGACTGAGCAGCATGACATGGCGGCCGCT |
| RBCS4T_R | CTATGATCCCCCGTGCAGAC |
| AATP1P_F | TAGCGGTCTCAGTGGTGGTA |
| AATP1P_R | GTCAGCTTGGCCATGATTCCTAGCCTGAAGGGTTAC |
| AATP1T_F | GAGGAGCAGGACTGAGCAGCCCGGCAGAGCGCTTGAC |
| AATP1T_R | GATAGGTTGTTGTTGGCGGC |
| ble_F | ATGGCCAAGCTGACCAGCGCCGT |
| ble_R | TCAGTCCTGCTCCTCGGCCACGA |
| neo_F | ATGGGATCGGCCATTGAACAAG |
| neo_R | TCAGAAGAACTCGTCAAGAAG |
| Pkneo_F | ATGGGCAGCGCTATTGAGCAG |
| Pkneo_R | TTAGAAGAACTCGTCCAGGAG |
| For the analysis of CRISPR/Cas9 target sites | |
| CDMT1_F | AGTCCTGGCCTCTGCATCTA |
| CDMT1_R | CGTAGGGCTTGAAGGTGACA |
| DMAN1_F | GGAACCTGTTGAACGAGCCT |
| DMAN1_R | TCAGTTTCGCGGATGTTCGA |
| DMAN1_R2 | AAGCCAAACCCTCTCAGACG |
| AATPL1_F | GCTTTGGCCTGCTCTATCCCTCTC |
| AATPL1_R | ACACACGAACCACCACCCTCTCTC |

**Additional table 2**

**crRNA sequences**

| Gene | Target | crRNA sequence (5'to 3') | GC % | Tm °C | Hit-12mer +PAM |
| --- | --- | --- | --- | --- | --- |
| *PkDMAN1* | 1 | AGUGGUGACCGACUGGUACCUGG | 60 | 77.7 | 2 |
|  | 2 | CCACAUGAUCACCACCGGAGAGG | 60 | 75.5 | 1 |
|  | 3 | AACGACAGCCUCAGCAACGGUGG | 60 | 77.2 | 1 |
| *PkAATPL1* | 1 | GGCCUUGCACCUCAAUGGGAUGG | 60 | 78.5 | 1 |
|  | 2 | CAACGAGACGACGUCCAUCGAGG | 60 | 73.1 | 1 |
|  | 3 | AACGUGGCUCAGACGAUGGCAGG | 60 | 77.4 | 1 |
| *PkCDMT1* | 1 | CCACCAAGACCAAGACUGACGCC | 60 | 75.9 | 1 |
|  | 2 | CCUACCUUCGCCACCGAGAUGUC | 60 | 76.8 | 1 |
|  | 3 | CACCGAGAUUGGCCAUGCCCGGG | 65 | 79.5 | 1 |

Hit-12 mer +PAM: potential off-target sites for each of the designed crRNAs were searched in the genome of strain NIES-2152 as perfectly matched 12-mer sequences adjacent to the PAM sequence to the 3’-end 12-mer sequence of the crRNA sequence. The number of off-target sites detected using using the CRISPRdirect software is indicated.

**Additional table 3**

**Statistical significance of the difference in growth- and lipid yields between the wild-type strain and other strains or between strain PK4 and other strains grown under continuous light conditions**

|  | day | Volumetric growth yield | | | | Lipid content | | | | Volumetric lipid yield | | | |
| --- | --- | --- | --- | --- | --- | --- | --- | --- | --- | --- | --- | --- | --- |
|  |  | WT | PK4 | CR12 | CR97 | WT | PK4 | CR12 | CR97 | WT | PK4 | CR12 | CR97 |
| vs WT | 4 |  | **–** |  |  |  |  | **+** | **+** |  | **−** |  |  |
|  | 7 |  | **− −** |  |  |  |  | **+** | **+** |  |  | **+** |  |
|  | 11 |  | **− −** |  |  |  |  | **+** | **+** |  | **−** |  |  |
|  | 14 |  | **− −** |  |  |  | **++** | **+** | **+** |  | **−** |  |  |
| vs PK4 | 4 | **+** |  | **+** | **+** |  |  | **+** | **+** | **+** |  | **+** | **+** |
|  | 7 | **++** |  | **++** | **++** |  |  |  |  |  |  | **+** | **+** |
|  | 11 | **++** |  | **++** | **++** |  |  |  |  | **+** |  | **+** | **+** |
|  | 14 | **++** |  | **++** | **++** | **− −** |  |  |  | **+** |  | **+** |  |

Strains PK4, CR12 and CR97 are defective in *AATPL1*.

+, −: P-value between 0.01 and 0.05; ++, − −: P < 0.01.

+ and – symbols indicate significantly higher and lower value than the control.

**Additional table 4**

**Statistical significance of the difference in growth- and lipid yields between wild-type and other strains or between strain PK4 and other strains grown under the L/D 10:14 cycle**

|  | day | Volumetric growth yield | | | | Lipid content | | | | Volumetric lipid yield | | | |
| --- | --- | --- | --- | --- | --- | --- | --- | --- | --- | --- | --- | --- | --- |
|  |  | WT | PK4 | CR12 | CR97 | WT | PK4 | CR12 | CR97 | WT | PK4 | CR12 | CR97 |
| vs WT | 4 |  | **− −** |  |  |  | **++** |  |  |  | **−** |  | **−** |
|  | 7 |  | **− −** |  |  |  |  | **+** | **+** |  | **−** |  |  |
|  | 11 |  | **−** |  |  |  | **+** | **+** | **++** |  |  | **+** | **+** |
|  | 14 |  | **−** |  |  |  | **++** | **+** | **++** |  |  | **+** | **++** |
| vs PK4 | 4 | **++** |  | **++** | **++** | **− −** |  | **++** | **++** |  |  |  |  |
|  | 7 | **++** |  | **++** | **++** |  |  |  | **+** |  |  |  |  |
|  | 11 | **+** |  | **+** | **+** | **−** |  |  |  |  |  |  | **+** |
|  | 14 | **+** |  | **+** | **+** | **− −** |  |  |  |  |  |  | **+** |

Strains PK4, CR12 and CR97 are defective in *AATPL1*

+, −: P-value between 0.01 and 0.05; ++, − −: P < 0.01.

+ and – symbols indicate significantly higher and lower value than the control.

**Additional table 5**

**Statistical significance of the difference in growth- and lipid yields between wild-type and other strains or between strain PK4 and other strains grown under the L/D 16:8 cycle**

|  | day | Volumetric growth yield | | | | Lipid content | | | | Volumetric lipid yield | | | |
| --- | --- | --- | --- | --- | --- | --- | --- | --- | --- | --- | --- | --- | --- |
|  |  | WT | PK4 | CR12 | CR97 | WT | PK4 | CR12 | CR97 | WT | PK4 | CR12 | CR97 |
| vs WT | 4 |  | **− −** |  |  |  |  |  |  |  |  |  |  |
|  | 7 |  | **− −** |  |  |  |  | **++** | **+** |  |  | **++** | **+** |
|  | 11 |  |  |  | **+** |  | **+** | **++** | **+** |  |  | **+** | **+** |
|  | 14 |  |  |  | **++** |  |  | **++** | **++** |  |  | **+** | **++** |
| vs PK4 | 4 | **++** |  | **++** | **++** |  |  |  |  |  |  | **+** |  |
|  | 7 | **++** |  | **++** | **++** |  |  |  |  |  |  | **+** |  |
|  | 11 |  |  |  | **+** |  |  |  |  |  |  |  |  |
|  | 14 |  |  | **+** | **++** |  |  |  |  |  |  | **+** | **+** |

Strains PK4, CR12 and CR97 are defective in *AATPL1.*

+, −: P-value between 0.01 and 0.05; ++, − −: P < 0.01.

+ and – symbols indicate significantly higher and lower value than the control.

**Additional table 6**

**Statistical significance of the difference in growth- and lipid yields between wild-type and other strains or between strain PK4 and other strains grown under the L/D 12:12 cycle**

|  | day | Volumetric growth yield | | | | Lipid content | | | | Volumetric lipid yield | | | |
| --- | --- | --- | --- | --- | --- | --- | --- | --- | --- | --- | --- | --- | --- |
|  |  | WT | PK4 | CR12 | CR97 | WT | PK4 | CR12 | CR97 | WT | PK4 | CR12 | CR97 |
| vs WT | 4 |  | **− −** |  |  |  |  |  |  |  |  |  |  |
|  | 7 |  | **−** |  |  |  |  |  | **++** |  |  |  | **+** |
|  | 11 |  |  |  |  |  | **++** | **+** | **++** |  | **+** | **+** | **++** |
|  | 14 |  |  |  |  |  | **++** | **++** | **++** |  | **+** | **+** | **+** |
| vs PK4 | 4 | **++** |  | **+** | **++** |  |  |  |  |  |  |  |  |
|  | 7 | **+** |  | **++** | **+** |  |  |  | **++** |  |  |  | **+** |
|  | 11 |  |  |  |  | **− −** |  |  |  | **−** |  |  |  |
|  | 14 |  |  | **+** | **+** | **− −** |  |  |  | **−** |  |  |  |

Strains PK4, CR12 and CR97 are defective in *AATPL1*.

+, −: P-value between 0.01 and 0.05; ++, − −: P < 0.01.

+ and – symbols indicate significantly higher and lower value than the control.
